# Single-cell immunoblotting resolves estrogen receptor-α isoforms in breast cancer

**DOI:** 10.1371/journal.pone.0254783

**Published:** 2021-07-27

**Authors:** John J. Kim, Wenchuan Liang, Chi-Chih Kang, Mark D. Pegram, Amy E. Herr

**Affiliations:** 1 Department of Bioengineering, University of California Berkeley, Berkeley, CA, United States of America; 2 Division of Medical Oncology, Department of Medicine, Stanford University, Stanford, CA, United States of America; Roswell Park Cancer Institute, UNITED STATES

## Abstract

An array of isoforms of the nuclear estrogen receptor alpha (ER-α) protein contribute to heterogeneous response in breast cancer (BCa); yet, a single-cell analysis tool that distinguishes the full-length ER-α66 protein from the activation function-1 deficient ER-α46 isoform has not been reported. Specific detection of protein isoforms is a gap in single-cell analysis tools, as the de facto standard immunoassay requires isoform-specific antibody probes. Consequently, to scrutinize hormone response heterogeneity among BCa tumor cells, we develop a precision tool to specifically measure ER-α66, ER- α46, and eight ER-signaling proteins with single-cell resolution in the highly hetero-clonal MCF-7 BCa cell line. With a literature-validated pan-ER immunoprobe, we distinguish ER-α66 from ER-α46 in each individual cell. We identify ER-α46 in 5.5% of hormone-sensitive (MCF-7) and 4.2% of hormone-insensitive (MDA-MB-231) BCa cell lines. To examine whether the single-cell immunoblotting can capture cellular responses to hormones, we treat cells with tamoxifen and identify different sub-populations of ER-α46: (i) ER-α46 induces phospho-AKT at Ser473, (ii) S6-ribosomal protein, an upstream ER target, activates both ER-α66 and ER-α46 in MCF-7 cells, and (iii) ER-α46 partitions MDA-MB-231 subpopulations, which are responsive to tamoxifen. Unlike other single-cell immunoassays, multiplexed single-cell immunoblotting reports–in the same cell–tamoxifen effects on ER signaling proteins and on distinct isoforms of the ER-α protein.

## Introduction

The estrogen receptor-α (ER-α66, Uniport P03372) is a steroid receptor expressed or overexpressed in ~75% of breast cancers (BCa) [1–4]. To block ER-α66 overexpression, adjuvant hormone therapies including tamoxifen (TAM) are used. TAM is a nonsteroidal-triphenylethylene selective estrogen receptor modulator (SERM) that was structurally derived from diethylstilbestrol-like estrogens and antiestrogens [5–7]. TAM mediates canonical ER signaling action, in which ER-α66 binds to estrogen response element (ERE) sites in DNA, thereby triggering transcription of estrogen-dependent genes [8]. However, BCa is a heterogeneous disease such that classification based on nuclear ER-α66 may be insufficient for hormone therapy selection [9]. Based on the Early Breast Cancer Trialists Group meta-analysis of 46,000 women who were disease-free after the first 5 years of hormone therapy, 21% of stage I patients had recurrence events at 20 years, 14% of which were distant metastasis [10–12]. Nearly all late-stage BCa patients develop clinical resistance to hormone therapies via a variety of mechanisms [13, 14].

A single-cell tool that discerns full-length and truncated ER-α isoforms may provide an insight for BCa response to hormone therapy. ER-α46 (46 kDa form of the 66 kDa full-length protein) is an alternatively spliced isoform with a missing activation function (AF-1) at the N-terminus. ER-α46 dimerizes with the full-length ER-α66 form to repress transcription [15–17]. Further, overexpression of ER-α46 has been observed to partially recover hormone sensitivity in hormone-insensitive BCa cell lines [18, 19].

Despite being implicated in hormone response, ER-α46 is difficult to distinguish from ER-α66 at the single-cell level. Widely used for biomarker discovery and cancer prognosis, protein microarrays and immunohistochemistry (IHC) [20, 21] identify cell-to-cell variation in oncoprotein expression. Because of homology between ER-α46 and ER-α66, isoform-specific antibodies are unable to distinguish ER-α46 from ER-α66 [16, 17]. Imaging mass cytometry offers subcellular resolution and target multiplexing (>30 protein), but like all immunoassays requires isoform-specific antibodies to distinguish ER-α46 from ER-α66 [22, 23]. Slab-gel immunoblotting resolves protein targets by differences in molecular mass and immunoprobing with a pan-ER antibody. Given detection sensitivity limitations of slab-gel immunoblotting, pooling of cells is required for detection. Pooling of cells obscures sub-populations with protein expression differences. Consequently, a single-cell tool that offers sub-population resolution and multiplexing of ER signaling is needed [24].

Here, we develop a single-cell immunoblotting that classifies BCa subtypes based on 10 protein targets involved in ER signaling, including the challenging separation of ER-α66 and ER-α46 isoforms, as described above. Seeking to validate single-cell detection of clonal subpopulations, we follow the studies of Leung, et al. [25] and Nugoli, et al. [26] and scrutinize the BCa cell line MCF-7 owing to expected high hetero-clonality and genetic plasticity. We utilize the monoclonal pan-ER (SP-1, C-terminal domain) antibody–tested in BCa cell lines [27, 28], mouse models [29], patient tumor ER-α status [30, 31]–as an immunoreagent to detect the frequency and expression levels of ER-α isoforms. As a negative control cell line lacking the ER-α isoforms, we follow published studies [32, 33] and employ the human embryonic kidney cell line HEK293. To study single-cell ER-α protein changes, we treat cells with either E2 or TAM. Like 4-hydroxytamoxifen, TAM is a nonsteroidal antiestrogen that binds to ER at a low affinity of dissociation constant at 4.8 nM and inhibits cell growth at 10 μM [34, 35]. Following the ligand treatment, we investigate BCa subpopulations based on the hormone response. The protein target multiplexing and isoform specificity offered by single-cell immunoblotting is used to gain understanding of the predictive potential of ER-α isoforms in heterogeneous BCa cells.

## Materials and methods

### Fabrication of open microfluidic devices using SU-8 soft lithography

A polyacrylamide gel was polymerized against a silicon wafer with SU-8 micropillars for microwells. After cleaning a mechanical grade silicon wafer (University Wafers) surface with isopropanol and acetone, a 30 μm SU-8 3050 (Y311075; MicroChem) layer was coated by spinning at 4000 RPM for 30 s and soft baked at 95°C for 15 min. Then, the wafer was soft baked at 95°C for 15 min, and exposed to UV (40 mW/cm^2^, 5 s) under a Mylar mask with the microwell array design (250 μm well-to-well spacing and 1.5 mm long separation lane). Followed by post-exposure baking (65°C for 1 min, 95°C for 5 min), the wafer was immersed in SU-8 developer (Y020100; Microchem) to reveal the micropillars. Before casting a polyacrylamide gel, the wafer was coated with 100 μl hydrophobic dichlorodimethylsilane (DMDCS, 440272; Sigma-Aldrich) via vapor-deposition for 40 min under vacuum. The SU-8 mold’s thickness was measured by using a surface profilometer (Sloan Dektak 3030) with a 0.10 mN stylus force. By casting on the SU-8 mold, the 30-μm polyacrylamide gel layer with patterned microwells was chemically polymerized using 8% T, 3.45% C acrylamide/bis-acrylamide (40% wt/wt) solution (A7802; Sigma-Aldrich), 3 mM N-[3-[(3-Benzoylphenyl)-formamido]propyl] methacrylamide (BPMAC, PharmAgra Laboratories), 0.08% ammonium persulfate (APS, A3678; Sigma-Aldrich) and 0.08% N,N,N′,N′-tetramethylethylenediamine (TEMED, T9281; Sigma-Aldrich).

### Primary tissue dissociation

Primary human tissues, which were slowly frozen in fetal bovine serum (FBS) with 10% dimethyl sulfoxide. Our Institutional Review Board deemed the study to be “not human subjects research”, owing to the authors’ use of Stanford Tissue Bank tissues that: existed before the research began, were not collected by the authors, and were de-identified prior to receipt by the authors. The authors did not collect potentially identifying genetic information. Tissue information is listed in **S1 Table in S1 File**. After quickly thawing, tissues were diced and incubated in a solution of collagenase type 3 (3000 unit/mL; 07423; Stemcell Technologies) and DNAse type 1 (D4263-5VL; 100 Kunitz unit/μl; Sigma-Aldrich) at 37°C for 4 h. After digesting extracellular matrices, cell clumps were dissociated by a 40 μm cell strainer (352340; Corning). The dissociated cells were then resuspended with Hank’s Balanced Salt Solution (14025076; Thermo Fisher Scientific) with 2% FBS.

### Cell lines and cell culture

MCF-7, MDA-MB-231, HEK293 were obtained from the American Type Culture Collection (ATCC). HEK293 was cultured in Eagle’s Minimum Essential Medium (EMEM) (30–2003; ATCC) supplemented with 1% penicillin streptomycin (PS) and 10% FBS. MCF-7 and MDA-MB-231 were maintained in RPMI 1640 (11875–093; Thermo Fisher Scientific), supplemented with 1% PS and 10% FBS. All cell lines were incubated in a humidified incubator held at 37 ˚C under 5% CO_2_. All cell lines were authenticated and free of mycoplasma using short tandem repeat analysis by UC Berkeley Cell Culture Facility. To limit sub-culturing effect, cell lines at low passage numbers (< 20) after thaw were only used for study.

### Tamoxifen

Prior to ligand treatment, cells were incubated in phenol free RPMI1640 (11835030; Thermo Fisher Scientific) and charcoal stripped FBS (A3382101; Thermo Fisher Scientific) with 1% PS for 48 h. Like 4-hydroxytamoxifen, tamoxifen (TAM, T5648; Sigma-Aldrich) is a nonsteroidal antiestrogen that binds to ER and inhibits cell growth at a low affinity of dissociation constant at 4.8 nM [34, 35]. Thus, similar to previous literature protocols [36, 37], cells were treated with TAM with final concentration of 10 μM for 24 h. For negative control, cells were treated with 100% EtOH with equal volume as in the TAM treatment for 24 h. After the treatment, cells were detached from cell culture dish with 10 mM EDTA (AM9260G; Thermo Fisher Scientific) and proceeded with the single-cell immunoblotting.

### Single-cell immunoblotting procedure

A single-cell immunoblot device is composed of a 30-μm thick polyacrylamide gel (8%T, 2.7%C) patterned with an array of 30-μm diameter microwells on a standard microscope glass slide. Starting with a suspension of cells at 25,000 cells/ml in 1x PBS (10010023; Thermo Fisher Scientific), gravitational sedimentation (10 min) populates microwells with cells, typically at 1 cell/microwell occupancy. After carefully washing the single-cell immunoblot with 1x PBS, more than 94% of microwells containing cells are occupied with single cell as determined by brightfield microscopy (**S1 Fig in S1 File**). Next, cells were lysed *in situ* for 30 s by pouring 15 ml of chemical lysis buffer at 37°C. The chemical lysis buffer is comprised of 8 M Urea (U5378, Sigma Aldrich), 1% sodium dodecyl sulfate (SDS, L3771; Sigma Aldrich), 0.1% Triton X-100 (X100; Sigma Aldrich), 1x Tris-glycine (D6750; Sigma Aldrich). Following cell lysis, an electric field at 40 V/cm was applied across the single-cell immunoblot device, driving for protein polyacrylamide gel electrophoresis (PAGE) for 30 s. Immediately after PAGE, separated proteins were covalently bounded to the gel (via light-activated benzophenone) by applying UV (40 mW cm^-2^, 45 s, Lightningcure LC5; Hamamatsu). Then, the single-cell immunoblot was washed with 1x TBS with Tween 20 (TBST, 77500; Affymetrix) for 1 h prior to immunoprobing. For immunoprobing, 0.1 g/l of primary and secondary antibodies were diluted with 1x TBST with 2% BSA and probed the device for 3 h and 2 h, respectively. After each probing step, 1x TBST was used for washing for 1 h. Lastly, the device was dried and scanned with a fluorescence microarray scanner (GenePix 4300A; Molecular Devices).

### Antibody probes

Primary antibodies of α-actinin (6487; Cell Signaling), β-TUB (ab6046; Abcam), CD44 (3570; Cell Signaling), ER-α (SP-1; Sigma Aldrich), cleaved caspase 8 (9496; Cell Signaling), cJUN (60A8; Cell Signaling), Cyclin A (4656; Cell Signaling), EGFR (2232; Cell Signaling), GAPDH (Sab2500450; Sigma Aldrich), ER-β (51–7700; Thermo Fisher), Phospho-AKT(Ser473, 9231; Cell Signaling), p38 MAPK (8690; Cell Signaling), S6-ribosomal protein (Ser240/244) (5364; Cell Signaling) were immunoprobes for BCa cell lines. For dissociated cells from primary tissues, we assayed with total 8 protein markers including β-TUB and panCK (Z0622; Dako). First, β-TUB is used to distinguish cells from cell debris and empty microwells. Second, panCK is used to further differentiate BCa epithelial cells from other contaminant cells. Third, ER-α isoforms identified ER-α^+^ BCa cells. Finally, ER signaling protein markers (CD44, Cyclin A, p38 MAPK, pAKT, pS6) selected different ER-α^+^ BCa subpopulations present in a tumor. Since the ER-α has been discontinued at the time of this publication, we suggest that interested researchers consider the same monoclonal SP-1 antibody from Thermo Fisher Scientific (MA1-39540).

Anti-goat antibody with Alexa Fluor 555 (A-21432; Thermo Fisher Scientific), anti-mouse antibody with Alexa Fluor 594 (A-11032), and anti-rabbit antibody conjugated with Alexa Fluor 647 (A-21245) were used as secondary antibodies. Secondary antibodies to goat IgG pre-labelled with AlexaFluor 488 and 555 (A11055 and A21432), mouse IgG pre-labelled with AlexaFluor 488 (A21202), and rabbit IgG pre-labelled with AlexaFluor 488 and 647 (A21206 and A31573) were used as prepared by the vendor (Invitrogen). For slab-gel immunoblotting, secondary antibodies to goat (A15999), rabbit (31460), mouse (31430) IgG labelled with horseradish peroxidase (HRP) were used as prepared by the vendor (Thermo Fisher Scientific).

### Single-cell immunoblotting data and statistical analyses

Images were processed by applying a median filter with a 2-pixel radius and a threshold value of 50 (ImageJ). Protein peaks from the single-cell immunoblot were quantitated with in-house MATLAB scripts [38]. The peaks were fitted by Gaussian functions in MATLAB (R2016b) and processed by extracting Gaussian parameters for peak width, location, and area-under-curve for protein expression. The protein peaks with Gaussian fitting R^2^ ≥ 0.65 and signal-to-noise ratio (SNR) > 3 were analyzed [38].

For statistical comparison of single-cell expression level, Mann-Whitney test was used. Kruskal-Wallis test with Dunn’s multiple comparison test was used for > 2 mean comparison of the single-cell expression level. Unpaired t-test with Welch’s correction was used to compare the cell subpopulation frequencies. The level of significance (p) is 0.05. For correlation studies, we used Spearman’s correlation coefficients (⍴) with Dunn and Sidák correction and accounted correlations with the p value ≥ 0.05.

Principal component analysis in MATLAB (2016b) is used for the multivariate analysis of protein expression levels from the single-cell immunoblotting. MATLAB’s zscore function is applied to standardize the protein expression levels with a mean of 0 and a standard deviation of 1. MATLAB’s pca function is used to compute the principal component coefficients, scores, and variances. The 95% confidence ellipses are calculated by eigenvalue decomposition with two standard deviations.

## Results

**Fig 1:**
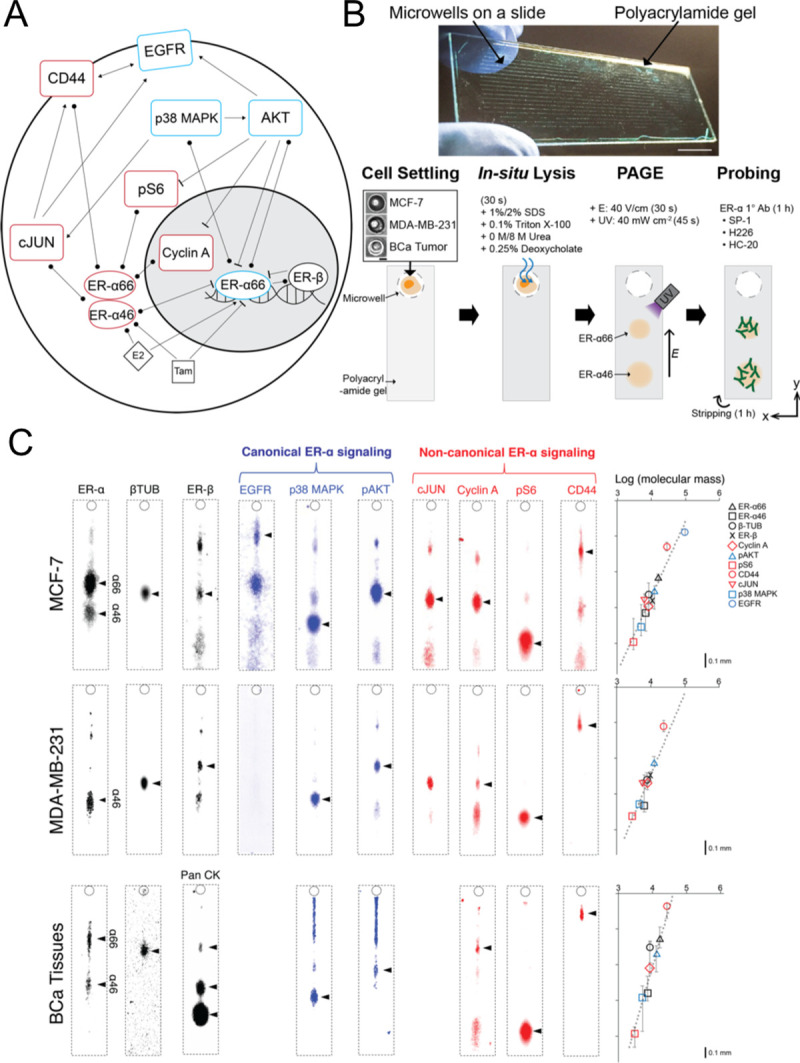
Single-cell immunoblotting resolves ER signaling proteins and ER-α isoforms each BCa cell. **(A)** Schematic of ER signaling, which includes crosstalk between canonical and non-canonical pathways. Network nodes indicate ER signaling proteins scrutinized. Canonical nodes in blue; non-canonical nodes in red. Edges represent protein-protein associations, arrows indicate activation, bars indicate inhibition, and circles represent an unspecified interaction. **(B)** Image and workflow of a single-cell immunoblotting device, which is a glass microscope slide layered with polyacrylamide gel (30-μm thick), stippled with an array of microwells (30-μm diameter). Schematic of assay workflow: after cells are settled into the microwells, each cell is chemically lysed (30 s) and the resultant single-cell lysate is separated by PAGE (40 V/cm, 30 s). Next, protein targets are photo-immobilized in the gel and interrogated with a sequence of antibody probes. **(C)** False-color fluorescence micrographs of single-cell immunoblots report both canonical and non-canonical ER signaling pathway targets in two relevant cell lines and cells dissociated from ER-α^3+^ BCa tumor (4318–1). Log-linear plots report molecular mass versus protein peak location and confirm protein target identity. Error bars represent the variance in protein target peak location, from a set of 10 single-cell immunoblots. Coefficient of determination for log-linear regression is R^2^_MCF-7, MDA-MB-231,BCa_Tissue_ = 0.9.

Exclusive reliance on nuclear overexpression of full-length ER-α66 as an indicator for hormone therapy may be insufficient [39–43]. The roles of truncated ER-α isoforms and non-canonical ER-α mechanisms are also important. Consequently, we investigated 10 distinct ER signaling proteins, related to canonical and non-canonical ER signaling pathways, at single-cell resolution (**Fig 1A**). We develop a single-cell immunoblot to scrutinize ER signaling and isoforms in hormone-sensitive (MCF-7) BCa, hormone-insensitive (MDA-MB-231) BCa, and patient-derived dissociated ER-α^3+^ BCa tumors (**Fig 1B and 1C**). As a model to detect clonal subpopulations with the single-cell immunoblot, MCF-7 was chosen as a cell line with high heteroclonality and genetic plasticity [25, 26]. Of note, HEK293 was used as a control cell line that lacks ER-α isoforms (**S2 Fig in S1 File**) [32, 33].

The single-cell immunoblot utilizes an open microfluidic device design (i.e., no enclosed microchannels or pneumatic control) to prepend single-cell polyacrylamide gel electrophoresis (PAGE) for size-based protein separation to an in-gel immunoassay (**Fig 1B**, **S3 Fig in S1 File**). As illustrated in **Fig 1C**, same-cell protein target multiplexing (up to 10 targets here) is achieved by immobilizing the separated proteins by UV, detecting with cocktails of compatible antibody probes, and thorough chemical stripping and re-probing of antibody probes for different protein targets [44].

To discern ER-α isoforms, we developed the single-cell immunoblotting by testing cell lysis conditions (SDS, urea) and several pan-ER-α antibodies in MCF-7, MDA-MB-231, and HEK293 cells (**S4 Fig in S1 File**). As corroborated by previous literature [27] and conventional assays (**S2 Fig in S1 File**), the SP-1 antibody identified ER-α isoforms without non-specific background signals in the single-cell immunoblot. After confirming molecular sizing with housekeeping proteins in in slab-gel and single-cell immunoblots (**S2**, **S4 Figs in S1 File**), we chose the monoclonal SP-1 antibody–widely used in cell lines [27, 28], mouse models [29], patient ER-α status [30, 31]–to investigate the frequency and expression levels of ER-α isoforms in BCa cell lines.

Given our interest in ER signaling, in addition to ER-α66 and ER-α46 isoform expression levels we perform single-cell immunoblotting for: EGFR, p38 MAPK, phospho-AKT at Ser473 (pAKT), cJUN, Cyclin A, phospho-S6 ribosomal protein (pS6), and CD44. For the non-canonical pathway, we examined the cJUN, Cyclin A, pS6, and CD44 protein targets, which lack ERE in their promoter regions (–10 kb to +5 kb from mRNA 5’-ends) [45, 46]. We also examined p38 MAPK and pAKT for the non-canonical pathway as these targets are reported to modulate ERE-independent and non-genomic ER signaling pathways [46–48].

### MCF-7 and MDA-MB-231 as BCa models for ER signaling

**Fig 2:**
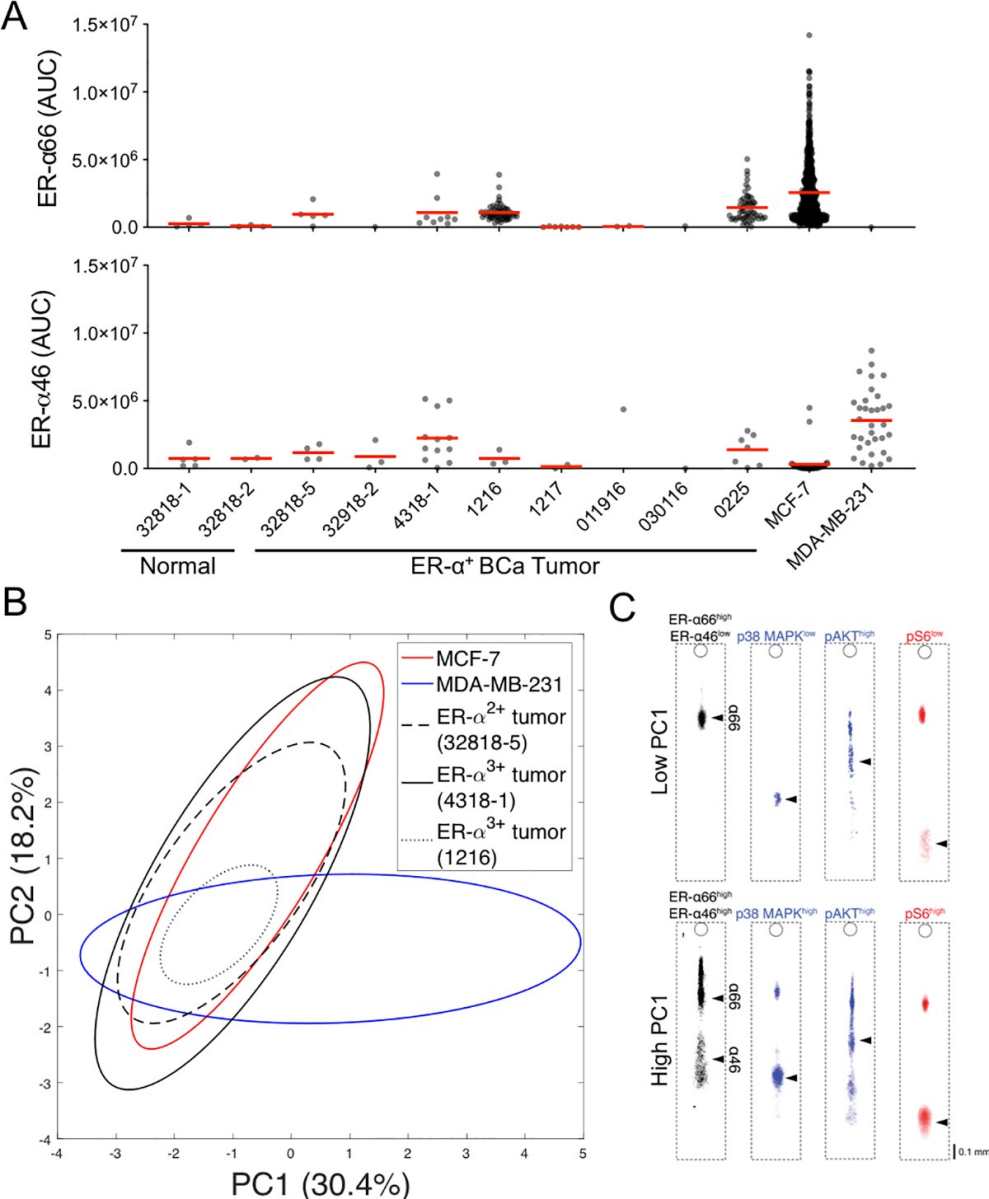
ER signaling in pilot cohort of patient-derived ER-α^+^ breast tumor cells is suitably modeled by both hormone-sensitive (MCF-7) and hormone-insensitive (MDA-MB-231) BCa cell lines. A Fluorescence quantitation (area-under-curve, AUC) of ER-α66 and ER-α46 expression levels in individual cells from 10 patient-derived breast tissue biopsies via single-cell immunoblotting shows tissue-to-tissue variation of ER-α66 and ER-α46 expression levels. Dots represent individual cells. Red lines represent mean values. B Unsupervised principal component analysis (PCA) finds a majority of the cells dissociated from 3 ER-α^+^ tumors converge with MCF-7 and MDA-MB-231 cells. Principal component 1 (PC1) and PC2 contribute to 30.4% and 18.2% variance in the marker expression level, respectively. MCF-7 (red) and MDA-MB-231 (blue) ellipses are plotted with 95% confidence interval of mean score. c Representative false-color fluorescence micrographs of 2 single-cell immunoblots in the ER-α^3+^ breast tumor (4318–1). Here, two sub-population expressing ER-signaling target expression levels to different degrees emerge: Cell Type 1 (low PC1, top) expresses low ER-α46, p38 MAPK, and pS6 expression levels. Cell Type 2 (high PC1, bottom) expresses high ER-α46, p38 MAPK, and pS6 expression levels.

Before we use the single-cell immunobloting to measure drug response in cell lines, we sought to understand how well hormone-sensitive (MCF-7) and hormone-insensitive (MDA-MB-231) BCa cell lines represent ER signaling, in comparison to patient-derived dissociated tissues (**Fig 1A, S1 Table in S1 File**). Single-cell immunoblotting detected both ER-α46 and ER-α66 in the dissociated specimens, including in cells from the normal breast tissues (**Fig 2A**). We observed that more than 3 ER-α^+^ tumor samples exhibited mean ER-α66 expression level (μ), measured in fluorescence area-under-curve, AUC (μ_ER-α66_32818–5_ = 0.97 × 10^6^, μ_ER-α66_4318–1_ = 1.09 × 10^6^, μ_ER-α66_1216_ = 1.09 × 10^6^, μ_ER-α66_0225_ = 1.46 × 10^6^; n_ER-α66_32818–5_ = 5 cells, n_ER-α66_4318–1_ = 9 cells, n_ER-α66_1216_ = 59 cells, n_ER-α66_0225_ = 55 cells; **Fig 2A**). Compared with cells dissociated from the ER-α^3+^ BCa tumor (0225), MCF-7 had a 1.3-fold higher expression level of ER-α66 (μ_MCF-7_ = 2.56 × 10^6^, μ_0225_ = 1.46 × 10^6^; n_MCF-7_ = 601 cells, n_0225_ = 55 cells; **Fig 2A**). In another case, the ER-α^3+^ BCa tumor (4318–1) had the highest mean ER-α46 expression level among ER-α^-/+^ breast tissue biopsies (μ_ER-α46,4318–1_ = 2.24 × 10^6^, n_ER-α46,4318–1_ = 12l cells), yet mean ER-α46 expression level in 4318–1 is 1.6-fold lower than mean ER-α46 expression level of MDA-MB-231 (μ_4318–1_ = 1.46 × 10^6^, μ_MDA-MB-231_ = 2.56 × 10^6^; n_4318-1_ = 55 cells, n_MDA-MB-231_ = 601 cells; **Fig 2A**).

Subsequently, we sought to compare variance in ER signaling between the ER-α^+^ BCa tumors and the two cell lines. For this investigation, we performed dimensional reduction on the multivariate analysis of the ER signaling protein levels (ER-α66, ER-α46, CD44, Cyclin A, p38 MAPK, pAKT, pS6). We tested whether the ER-α^+^ BCa tumors and the cell lines differ in the ER-signaling target expression level by carrying out principal component analysis (PCA). The first and second principal components (PC) explain the major variance (48.6%) of the ER-signaling target expression level (**Fig 2B**). Using the first and second principal components (PC), we investigated mean scores with confidence ellipses (**Fig 2B)**. The convergence of the 95% confidence ellipses between the ER-α^+^ BCa tumors and MCF-7 in the PC1 and PC2 score plot explains similarity between the ER-α^+^ breast tumors and MCF-in the variance of ER-signaling target expression level (**Fig 2B**). Conversely, we observe the 95% confidence ellipse of MDA-MB-231 diverging from the confidence ellipses of MCF-7 and ER-α^+^ breast tumors at PC1 > 2. As the PC1 score increases, positive correlations are found with ER-α46 (ρ_ER-α46_ = 0.30), p38 MAPK (ρ_p38_MAPK_ = 0.55), pAKT (ρ_pAKT_ = 0.45), and pS6 (ρ_pS6_ = 0.48) markers (**Fig 2C)**. For the PC2, ER-α66 (ρ_ER-α66_ = 0.69) and pAKT (ρ_pAKT_ = 0.51) are two dominant correlation coefficients. Consequently, the confidence ellipses in PCA of ER-signaling target expression level indicate similar variance in the ER-signaling pathway between the ER-α^+^ BCa tumors and the cell lines.

### Subpopulations of hormone-sensitive MCF-7 cells expressing either or both of ER-α66 and ER-α46

**Fig 3:**
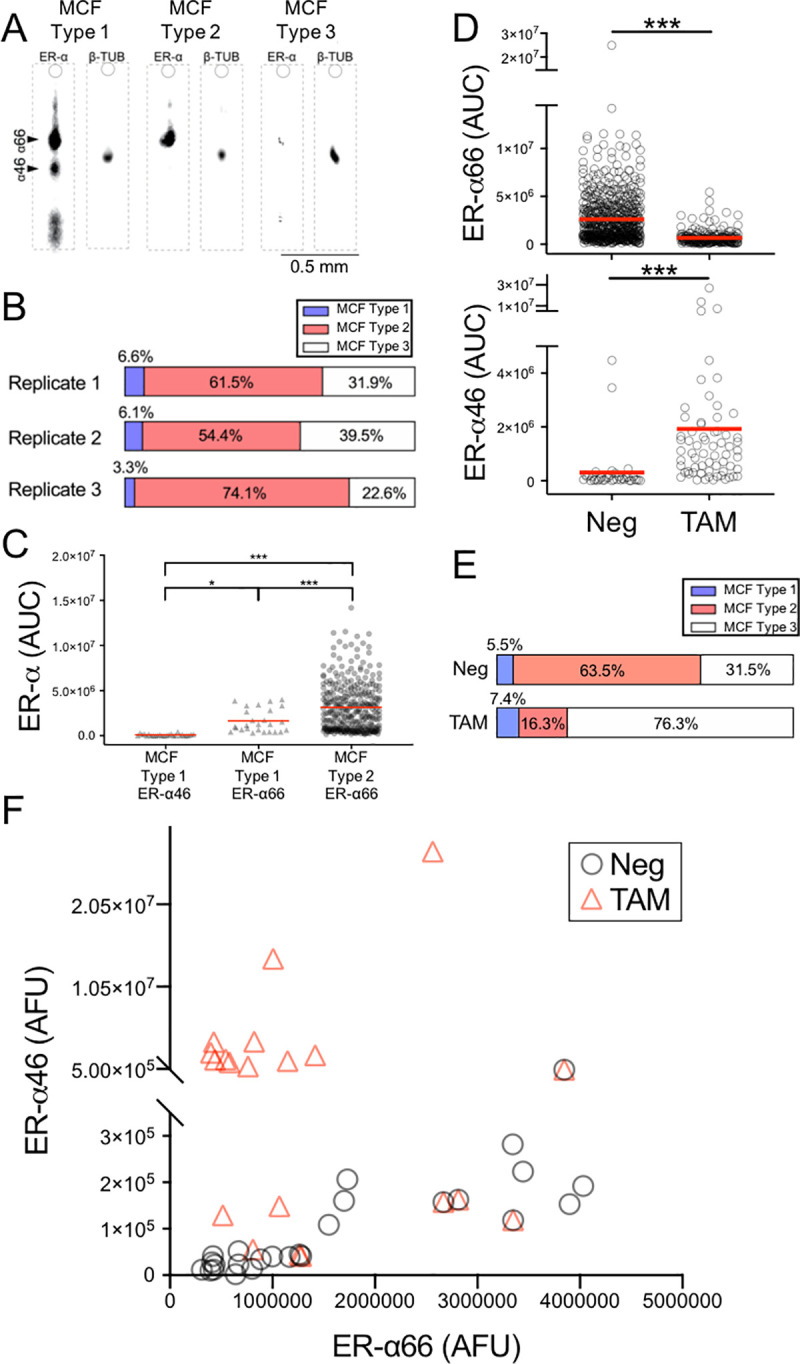
Hormone-sensitive BCa cells (MCF-7) comprise 3 subpopulations, distinguished by ER-α46 expression levels. **A** False-color micrographs of MCF-7 Cell Types: (1) ER-α66^+^ ∩ ER-α46^+^, (2) ER-α66^+^ ∩ ER-α46^-^, and (3) ER-α66^-^ ∩ ER-α46^-^. **B** Stacked bar graphs show ER-α subpopulation frequencies (3 single-cell immunoblot devices, n_1_ = 91 cells, n_2_ = 215 cells, n_3_ = 215 cells; 3 different populations). **C** Scatter plots of ER-α66 and ER-α46 expression levels in individual MCF-7 cells (*p = 0.03, ***p < 0.0001, n_ER-α66_only_ = 329 cells, n_both_ = 26 cells). Red lines represent mean values. **D** Top: scatter plots indicate that mean ER-α66 expression level is decreased in ER-α66^+^ subpopulation by TAM (***p < 0.0001, n_neg_ = 604, n_TAM_ = 227). Bottom: scatter plots show that TAM increases mean ER-α46 expression level (***p < 0.0001, **p< 0.005, n_neg_ = 38, n_TAM_ = 65). Red lines denote mean values. n_TAM_device1_ = 188 cells, n_TAM_device2_ = 204 cells, n_TAM_device3_ = 229 cells. **E** Stacked bar graphs show TAM repression on the MCF Cell Type 2 subpopulation frequencies (μ_neg_type2_ = 63.5, μ_TAM_type2_ = 16.3, ***p < 0.0001, n_neg_ = n_TAM_ = 3 devices) yet having no significant effects on the MCF Cell Type 1 frequency (μ_neg_type1_ = 5.5, μ_TAM_type1_ = 7.4, p > 0.3, n_neg_ = n_TAM_ = 3 devices). **F** Scatter plots of ER-α66 and ER-α46 expression levels in the MCF Cell Type 1 subpopulation indicate that cells treated with TAM express high ER-α46. ER-α66 expression level in the MCF Cell Type 1 subpopulation is reduced when cells are treated with TAM. Circles denote cells without treatment. Triangles denote cells treated with TAM (n_neg_ = 26 cells, n_TAM_ = 22 cells).

Next, we investigated ER-α isoform heterogeneity in the hormone-sensitive and hormone-insensitive cell lines by analyzing single-cell expression level and frequency (fraction of cells expressing ER-α isoforms out of the cell population). Single-cell immunoblotting analysis of hormone-sensitive BCa (MCF-7) cells revealed three distinct subpopulations: MCF Cell Type 1 expressing both ER-α isoforms (ER-α66^+^ ∩ ER-α46^+^; ∩ denotes intersection of two proteins), MCF Cell Type 2 with ER-α66^+^ ∩ ER-α46^-^, and MCF Cell Type 3 with ER-α66^-^ ∩ ER-α46^-^ (**Fig 3A**). On average, MCF-7 was composed of: MCF Cell Type 1 at 5.3% of the population (σ = 1.8%, n = 3 same-passage flasks), MCF Cell Type 2 at 63.2% of the population (σ = 9.8%, n = 3 same-passage flasks), and MCF Cell Type 3 at 31.3% of the population (σ = 8.51%, n = 3 same-passage flasks) (**Fig 3B**). Flow cytometry and slab-gel immunoblots corroborate the ER-α^+^ subpopulation frequency and relative expression levels, respectively (MCF Cell Types 1 and 2 = 74%, MCF Cell Type 3 = 26%; **S2, S4 Figs in S1 File**).

As compared to the MCF Cell Type 1 subpopulation, we measured a 2-fold higher mean ER-α66 expression level and associated CV in the MCF Cell Type 2 subpopulation (μ_Type2_ = 3.15 × 10^6^, CV_Type2_ = 84.5%, n_Type2_ = 329, compared to μ_Type1_ = 1.66 × 10^6^, CV_Type1_ = 77.6%, n_Type1_ = 26; **Fig 3C**). However, within the MCF Cell Type 1 subpopulation, we observe strong correlation of ER-α66 and ER-α46 at the basal level (Spearman’s correlation test, ⍴ = 0.96) (**Fig 3F)**, suggesting that protein expression of ER-α66 and ER-α46 might be mutually regulated at the basal level.

### Heterogeneous ER-α66 and ER-α46 response to TAM in hormone-sensitive (MCF-7) BCa

We next sought to understand how TAM affects ER-α66 and ER-α46 frequencies and mean expression levels. At the single-cell level, we hypothesize that TAM would reduce the ER-α66^+^ subpopulation. Since the TAM effect on ER-⍺46 is not known, we examined the ER-⍺46 expression level by using the single-cell immunoblots with TAM-treated cells.

In the hormone-sensitive BCa cells (MCF-7), as expected in the literature [49], the TAM-treated population showed a 4-fold decrease in ER-α66 expression level, as compared with basal ER-α66 expression level in the no-treatment group (μ_neg_ = 2.63 × 10^6^, μ_TAM_ = 0.68 × 10^6^, n_neg_ = 604 cells, n_TAM_ = 227, ***p < 0.0001, **Fig 3D**). Further, significant changes in the subpopulation frequencies for TAM-treated cells were observed. The mean MCF Cell Type 2 frequency of TAM-treated cells is significantly lower than the mean MCF Cell Type 2 frequency of non-treated cells (μ_TAM_ = 16.3%, μ_Neg_ = 63.5%, n_Neg_ = n_TAM_ = 3 devices, p < 0.0001; **Fig 3E**).

Interestingly, the mean frequency of MCF Cell Type 1 (ER-α66^+^ ∩ ER-α46^+^) in TAM-treated groups remained unaffected by TAM (μ_freq_TAM_ = 7.4%, μ_freq_neg_ = 5.5%). Instead, mean ER-α46 expression level increased in TAM-treated groups (μ_AUC_TAM_ = 1.94 × 10^6^, μ_AUC_neg_ = 0.31 × 10^6^; **Fig 3E and 3F**). In the MCF Cell Type 1 subpopulation, a strong correlation between ER-α66 and ER-α46 observed at the basal level (⍴_neg_ = 0.96) is lost in TAM-treated groups (⍴_TAM_ = -0.3; **Fig 3F**).

Narrowing our investigation to the MCF Cell Type 1 subpopulation, TAM significantly increased mean the ER-α46 expression level (μ_AUC_neg_ = 0.10 × 10^5^_,_ μ_AUC_TAM_ = 3.04 × 10^6^, n_neg_ = 26 cells, n_TAM_ = 20 cells, p_neg_TAM_ = 0.0001; **Fig 3F**), with no significant effect on the mean ER-α66 expression level (μ_AUC_neg_ = 1.67 × 10^6^_,_ μ_AUC_TAM_ = 0.85 × 10^6^, n_neg_ = 26 cells, n_TAM_ = 20 cells, p_neg_TAM_ = 0.28; **Fig 3F**. Although the ER-α66 expression level is lower in the subpopulation of cells expressing ER-α46 (versus subpopulations with no ER-α46 expression), TAM does not affect the ER-α66 expression level, when the ER-α46 isoform is expressed in that same cell. Taken together, the hormone (TAM) treatment significantly reduces the canonical ER-α66^+^ only cell subpopulation (MCF Cell Type 2), without affecting the ER-α66 in ER-α66^+^ ∩ ER-α46^+^ cell populations (MCF Cell Type 1). The observation suggests differential TAM treatment response in heterogeneous BCa.

Similar to TAM, we used the single-cell immunoblot to measure ER-α isoform expression and frequency after estradiol (E2) treatment **(S5 Fig in S1 File)**. The MCF Cell Type 2 subpopulation frequencies are significantly lower in the E2-treated cells than the non-treated cells (μ_E2_ = 24.7%, μ_neg_ = 68.5%; n_neg_ = n_E2_ = 3 devices, p < 0.0001; **S5 Fig in S1 File**). For the MCF Cell Type 1, the mean ER-α46 expression level increased in the E2-treated group (μ_AUC_E2_ = 3.20 × 10^6^, μ_AUC_neg_ = 0.31 × 10^6^, p < 0.05, **S5 Fig in S1 File**). Further, the strong correlation between ER-α66 and ER-α46 expression levels decreases in the E2-treated group (Pearson’s correlation test, ⍴_neg_ = 0.96, ⍴_E2_ = 0.17 **S5 Fig in S1 File**). At a high E2 concentration (1 μM), the decrease in subpopulation frequency of the MCF Cell Type 2 and the increase in ER-α46 expression level align with ER-α66 degradation via 26S proteasomal degradation pathways [50–53] and high ER-α46 transcription level [54] by E2, respectively.

### Rare subpopulation of hormone-insensitive BCa cells expresses ER-α46

**Fig 4:**
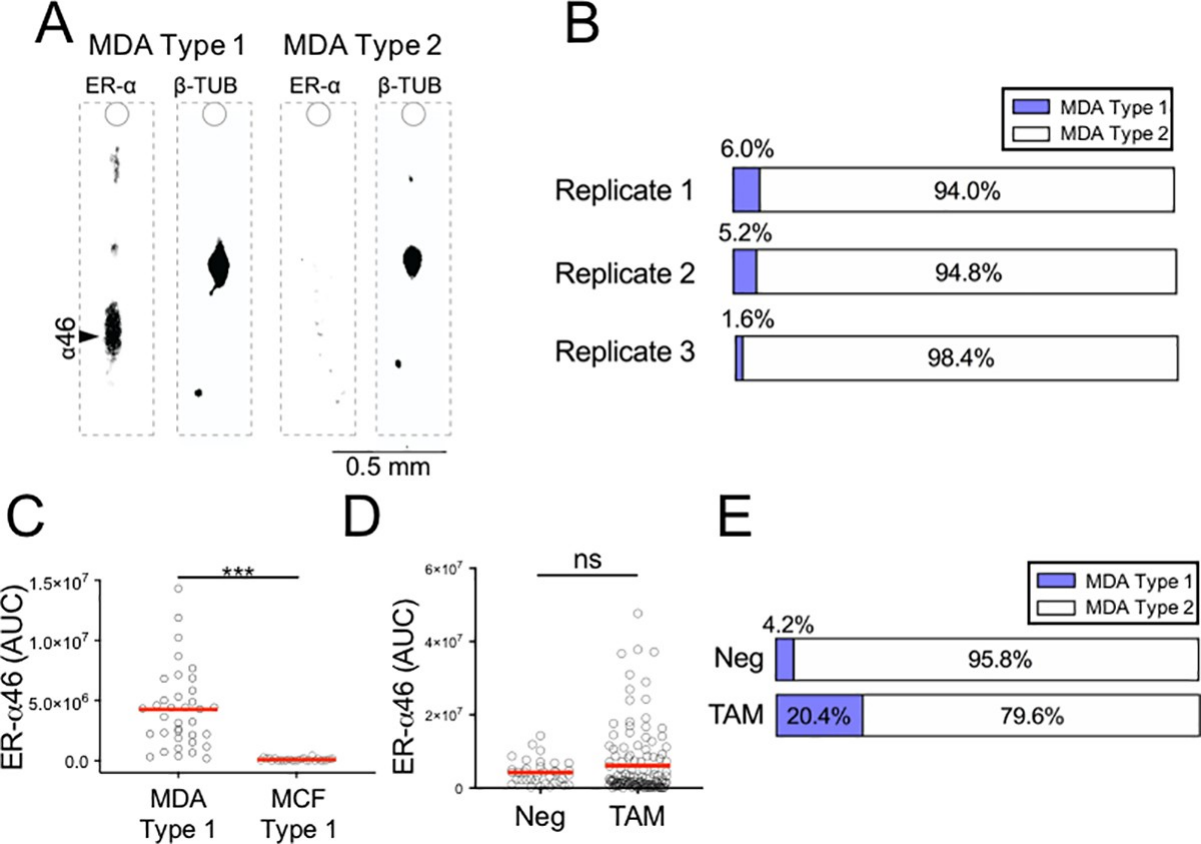
Hormone-insensitive BCa cells (MDA-MB-231) comprise 2 subpopulations, distinguished by ER-α46 expression levels. **A** False-color micrographs represent 2 cell subpopulations: ER-α46^+^, ER-α46^-^. **B** Stacked bar graphs show ER-α subpopulation frequencies in 3 single-cell immunoblot devices (n_1_ = 269 cells, n_2_ = 233 cells, n_3_ = 453 cells) from 3 different populations. **C** Scatter plot distributions of ER-α46 expression level in the MDA Cell Type 1 (MDA-MB-231) cell subpopulation compared to the MCF Cell Type 1 (MCF-7) cell subpopulation. (***p < 0.0001, n_type1_MDA-MB-231_ = 36 cells, n_MCF-7_ = 26 cells). Red lines denote mean values. **D** TAM affects ER-α46 expression level (*p = 0.03, n_neg_ = 36 cells, n_TAM_ = 109 cells). **E** Stacked bar graphs show MDA-MB-231 frequencies in response to TAM. TAM significantly increases the frequency of cells classified as the MDA Cell Type 1 subpopulation (*p = 0.04, n_neg_ = n_TAM_ = 3 devices). n_neg_device1_ = 269 cells, n_neg_device2_ = 233 cells, n_neg_device3_ = 453 cells; n_TAM_device1_ = 189 cells, n_TAM_device2_ = 214 cells, n_TAM_device3_ = 125 cells.

After characterizing ER-α isoforms in hormone-sensitive MCF-7 cells, we sought to understand the heterogeneity of ER-α46 expression level in a triple-negative BCa cell line, MDA-MB-231. The MDA-MB-231 cells lack the full-length ER-α66 protein and exhibit highly invasive phenotypes [55]. As expected, we did not detect ER-α66 in individual MDA-MB-231 cells using single-cell immunoblotting (**Fig 4A**).

Two cell subpopulations were identified: MDA Cell Type 1 with ER-α46^+^ and MDA Cell Type 2 with ER-α46^-^ (**Fig 4A**). The MDA Cell Type 1 subpopulation accounts for 4.2% (average of 3 replicates in **Fig 4B**) of the MDA-MB-231 cell population analyzed. Within the total cell population, the percentage of MDA Cell Type 1 (4.2%) is not significantly different from that of MCF Cell Type 1 (5.3%, average of 3 replicates in Fig 3B; n_MDA-MB-231_ = 3 devices; n_MCF-7_ = 3 devices; p = 0.91; **Figs 3B and 4B**). Interestingly, the mean ER-α46 expression level is 4-fold greater in the hormone-insensitive MDA-MB-231 cells, as compared to the hormone-sensitive MCF-7 cells (μ_MDA-MB-231_ = 4.26 × 10^6^, μ_MCF-7_ = 1.01 × 10^6^, n_MDA-MB-231_ = 36, n_MCF-7_ = 26, p < 0.0001; **Fig 4C**). The variance of the ER-α46 expression level was lower in the MDA-MB-231 cells (CV = 77.7%) than in the MCF-7 cells (CV = 105.3%), indicating less cell-to-cell variation in the ER-α46 expression level within the population of MDA-MB-231 cells (**Fig 4C**).

Next, we examined the ER-α46 expression level after TAM treatment in the MDA Cell Type 1 (ER-α46^+^) subpopulation of the hormone-insensitive MDA-MB-231 cells. In contrast to MCF-7 cells **(Fig 3E)**, we did not observe distinct TAM effects on the mean ER-α46 expression level in MDA-MB-231 cells (μ_AUC_neg_ = 4.30 × 10^6^, μ_AUC_TAM_ = 6.20 × 10^6^, n_neg_ = 36 cells, n_TAM_ = 109 cells, p_neg_TAM_ = 0.30, **Fig 4D**). However, the mean frequency of the MDA Cell Type 1 subpopulation increased in response to TAM (μ_Freq_neg_ = 4.2%_,_ μ_Freq_TAM_ = 20.4%, n_neg_ = n_TAM_ = 3 devices, p_neg_TAM_ = 0.04; **Fig 4E**). Similar to TAM, the E2 treatment had no significant effect on the mean ER-α46 expression (μ_AUC_neg_ = 4.30 × 10^6^, μ_AUC_E2_ = 3.20 × 10^6^, n_neg_ = 36 cells, n_E2_ = 50 cells, p_neg_E2_ = 0.06, **S5 Fig in S1 File**). Indeed, MDA-MB-231 is a hormone-insensitive BCa that the mean ER-α46 expression level does not change after the TAM or E2 treatments [56]. However, the single-cell immunoblotting uniquely detects the increase in the mean frequency of ER-α46^+^ after the TAM treatment.

### ER signaling proteins are highly correlated in TAM-treated hormone-sensitive and hormone-insensitive BCa cells

To evaluate whether TAM affects both canonical and non-canonical ER actions [57], we assessed associations between ER-α isoforms and ER signaling proteins in hormone-sensitive MCF-7 cells and hormone-insensitive MDA-MB-231 cells. We sought to investigate the canonical ER signaling response by measuring EGFR, p38 MAPK, and phospho-AKT (pAKT, phosphorylation at Ser473) protein targets, which are translated from genes enriched with ERE [45, 46], while CD44, pS6, and Cyclin A for non-canonical ER signaling.

**Fig 5:**
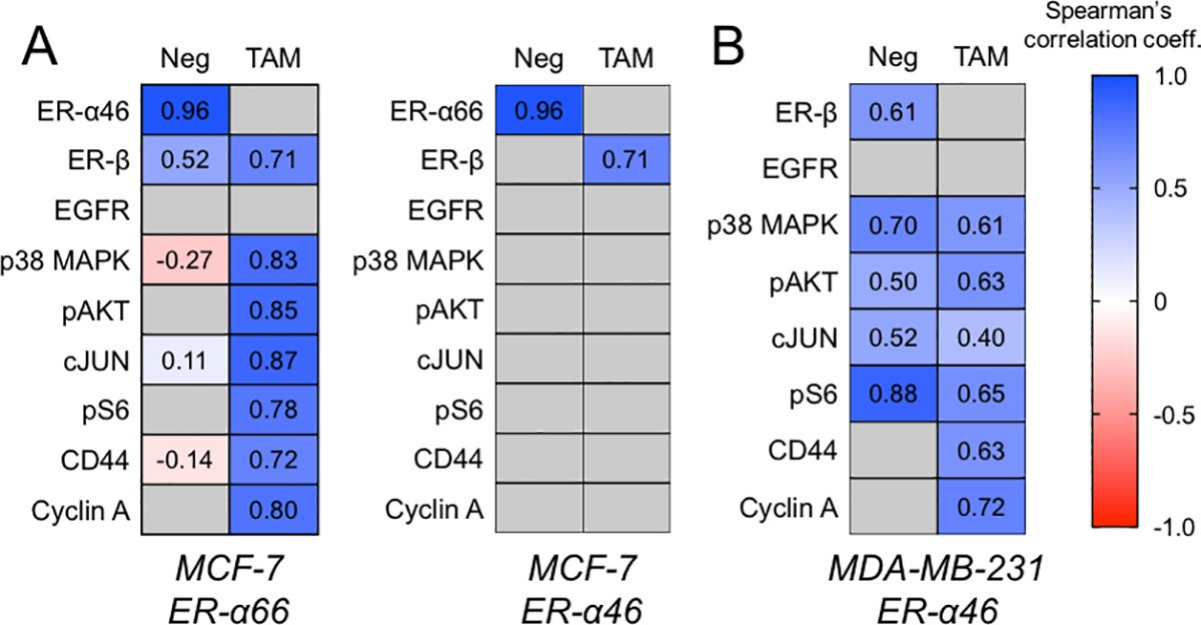
TAM treatment affects interactions between ER-α isoforms and ER signaling proteins. **A** Left: correlation matrix of full-length ER-α66 and ER signaling proteins in hormone-sensitive MCF-7 cells, with Spearman’s correlation values. Right: correlation matrix of ER-α46 and ER signaling proteins in hormone-sensitive MCF-7 cells, with Spearman’s correlation values. **B** Correlation matrix of ER-α46 and ER signaling proteins in hormone-insensitive MDA-MB-231 cells, with Spearman’s correlation values. Grey shading denotes correlation with p > 0.05.

At a basal level, the full-length ER-α66 protein is associated with the ER-α46 protein (⍴ = 0.96) and the ER-β protein (⍴ = 0.52, **Fig 5A**). With TAM treatment, while we did not observe significant changes in protein expression of ER-signaling targets with the exception of pAKT (**S2 Table in S1 File**). We observed strong correlation of ER-α66 with p38-MAPK (⍴ = 0.83), Cyclin A (⍴ = 0.80), cJUN (⍴ = 0.87), ER-β (⍴ = 0.71), pS6 (⍴ = 0.78), CD44 (⍴ = 0.72), pAKT (⍴ = 0.85; **Fig 5A**). In contrast, ER-α46 is less correlated (< 0.4) with any ER signaling targets in the TAM-treated group (**Fig 5A**). Taken together, in hormone-sensitive MCF-7, TAM reduces ER-α66 isoform expression but activates both canonical and non-canonical pathway (**Fig 5A**).

Since the mean frequency of ER-α46 positive subpopulation in hormone-insensitive MDA-MB-231 cells are induced by TAM **(Fig 4D and 4E**), we expect that TAM alters the strength of the relationship between ER-α46 and ER signaling proteins. At the basal level, ER-α46 is correlated with p38-MAPK (⍴ = 0.70), cJUN (⍴ = 0.52), ER-β (⍴ = 0.61), pS6 (⍴ = 0.88), and pAKT (⍴ = 0.50; **Fig 5B**). Interestingly, compared to the basal level, TAM decreases the correlation of ER-α46 with p38-MAPK (⍴ = 0.61), cJUN (⍴ = 0.40), and pS6 (⍴ = 0.65) while establishing a new correlation with Cyclin A (⍴ = 0.72) and CD44 (⍴ = 0.63; **Fig 5B**).

### pAKT is a key regulator of TAM sensitivity in the ER signaling pathway

**Fig 6:**
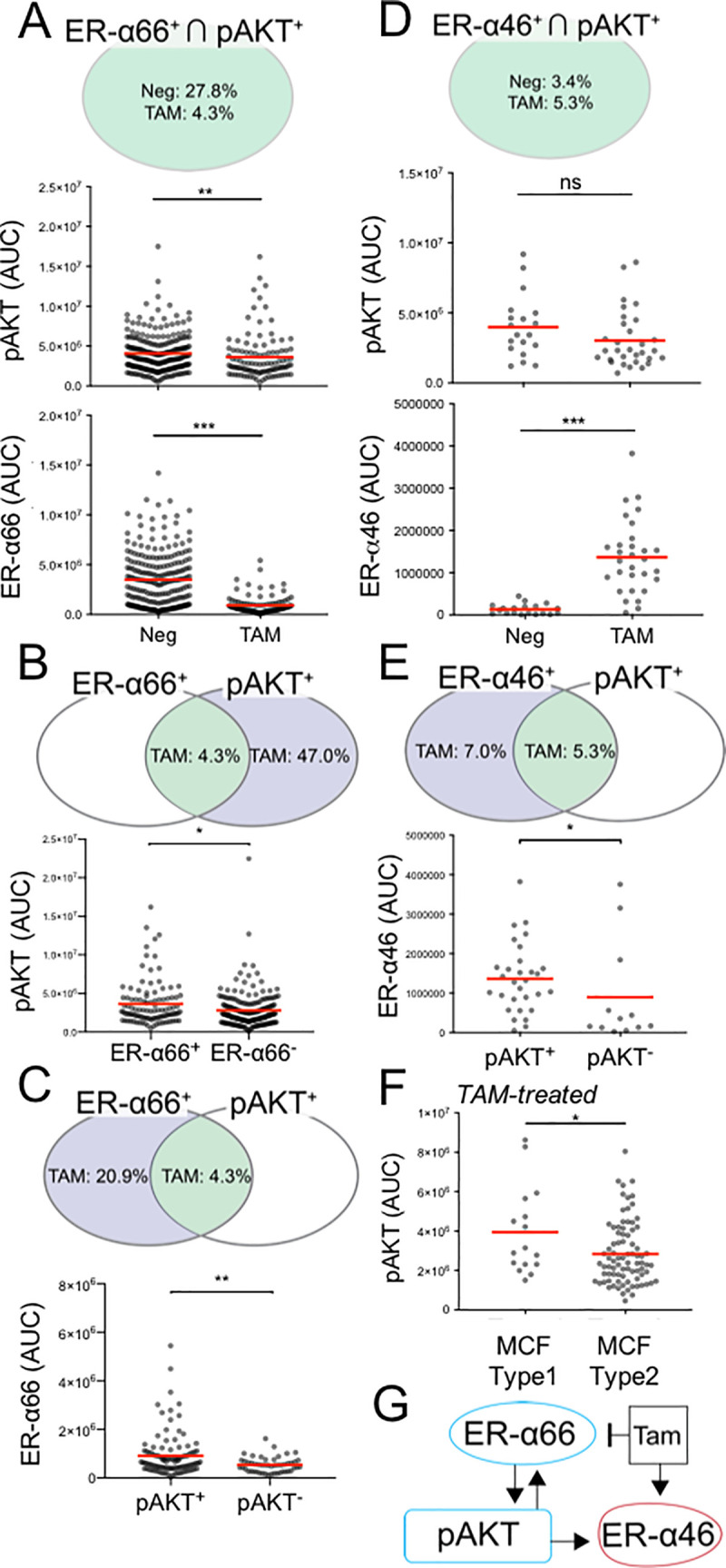
Subpopulation analysis reveals mutual activation between pAKT and ER-α isoforms in hormone-sensitive MCF-7 cells. **A** Venn diagram represents mean ER-α66^+^ ∩ pAKT^+^ subpopulation frequencies. Scatter plots show TAM effects on the ER-α66^+^ ∩ pAKT^+^ subpopulation. **B** Venn diagram represents mean subpopulation frequencies of ER-α66^+^ ∩ pAKT^+^ (green) and ER-α66^-^ ∩ pAKT^+^ (blue) in TAM. Scatter plots show different TAM effects on pAKT between the subpopulations with and without ER-α66. **C** Venn diagram represents mean subpopulation frequencies of ER-α66^+^ ∩ pAKT^+^ (green) and ER-α66^+^ ∩ pAKT^-^ (blue) in TAM. Scatter plot compares TAM effect on ER-α66 between the ER-α66^+^ ∩ pAKT^+^ and ER-α66^+^ ∩ pAKT^-^ subpopulations. **D** Venn diagram represents mean ER-α46^+^ ∩ pAKT^+^ subpopulation frequencies. Scatter plots show the TAM effects on the ER-α46^+^ ∩ pAKT^+^ subpopulation. No significant effects on mean single-cell expression of pAKT (p_Neg vs. TAM_ = 0.17, **S3 Table in S1 File**). **E** Venn diagram represents mean subpopulation frequencies of ER-α46^+^ ∩ pAKT^+^ (green) and ER-α46^+^ ∩ pAKT^-^ (blue) in TAM. Scatter plot compares TAM effect on ER-α66 between the ER-α46^+^ ∩ pAKT^+^ and ER-α46^+^ ∩ pAKT^-^ subpopulations. **F** Scatter plot compares TAM effect on pAKT between the MCF Cell Type 1 (ER-α66^+^ ∩ ER-α46^+^) and the MCF Cell Type 2 (ER-α66^+^ ∩ ER-α46^-^). **G** Diagram highlights TAM activation on ER-α46 and repression on ER-α66. The uni-directional (pAKT to ER-α46) and mutual (pAKT and ER-α66) activation are depicted. Red lines in scatter plots denote mean values. Detailed statistical tests are reported in **S3, S4 Tables in S1 File** (*p < 0.05, **p < 0.01, ***p < 0.0001, ns: p > 0.05).

We further investigated the relationship between ER-α isoform and ER signaling proteins by analyzing expression levels in each subpopulation. Since pAKT interacts both upstream and downstream in ER signaling pathways [58], we hypothesized that pAKT and ER-α isoforms would influence each other in TAM-treated hormone-sensitive BCa (MCF-7). In the ER-α66^+^ ∩ pAKT^+^ subpopulation, the mean pAKT expression level is decreased by 11.3% in TAM-treated group (**Fig 6A, S3 Table in S1 File**). Further, TAM lowered the mean ER-α66 expression level by 73.9% in the ER-α66^+^ ∩ pAKT^+^ subpopulation (**Fig 6A, S3 Table in S1 File**). In comparing the ER-α66^+^ ∩ pAKT^+^ and the ER-α66^-^ ∩ pAKT^+^ subpopulations, the ER-α66^+^ ∩ pAKT^+^ subpopulation has a mean pAKT expression level that is 22.2% lower in the ER-α66^-^ ∩ pAKT^+^ subpopulation (**Fig 6B, S4 Table in S1 File**). Reciprocally, TAM repression on ER-α66 expression level is lower in the pAKT^+^ than in the pAKT^-^ subpopulations (**Fig 6C, S4 Table in S1 File**).

Next, we sought to understand the relationship between ER-α46 and pAKT. We clustered the ER-α46^+^ ∩ pAKT^+^ MCF-7 cell subpopulation and measured responses to TAM treatment. Unlike the pAKT expression level at the population level (**S6 Fig, S2 Table in S1 File)**, we did not observe repression in the mean pAKT expression level within the ER-α46^+^ ∩ pAKT^+^ subpopulation upon TAM treatment **(Fig 6D, S3 Table in S1 File)**. We observed that the pAKT expression level is higher in the MCF Cell Type 1 (ER-α66^+^ ∩ ER-α46^+^) than in the MCF Cell Type 2 (ER-α66^+^ ∩ ER-α46^-^) subpopulation **(Fig 6F)**; however, the difference in pAKT expression is attributable to a greater decrease in ER-α66^+^ in the MCF Cell Type 2 (vs. MCF Cell Type 1) subpopulation and may not be associated with ER-α46 expression. Taken together, we suspect that the non-canonical ER signaling action of TAM is linked with the ER-α66 isoform (and not the ER-α46 isoform) via the pAKT signaling pathway in MCF-7 hormone sensitive cells (**Fig 6G**).

### p38 MAPK is associated with ER-α66 upon TAM treatment

**Fig 7:**
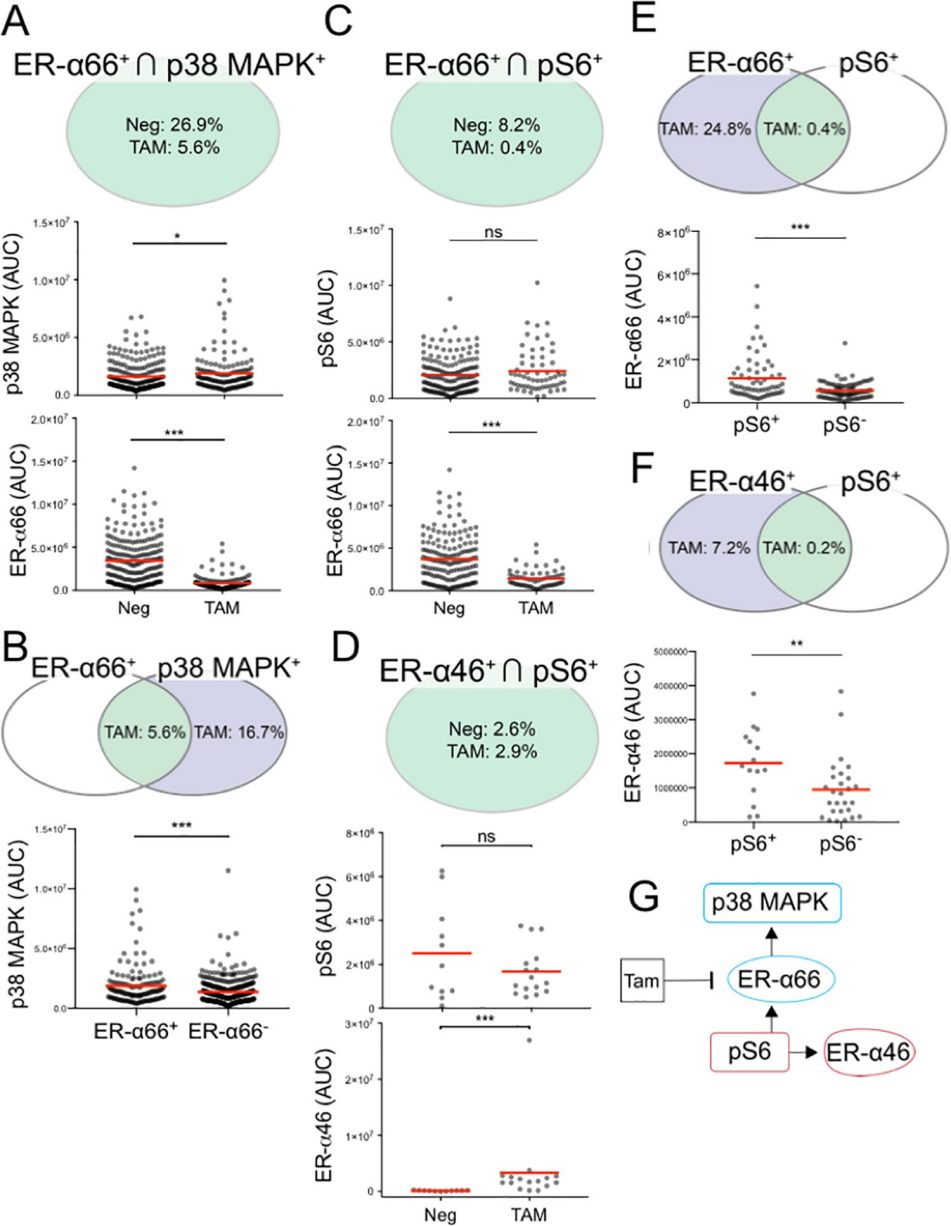
Subpopulation analysis uncovers ER-α66 activation on p38 MAPK and pS6 activation on both ER-α66 and ER-α46 in MCF-7 cells. **A** Venn diagram represents mean ER-α66^+^ ∩ p38 MAPK^+^ subpopulation frequencies. Scatter plots show TAM effects on the ER-α66^+^ ∩ p38 MAPK^+^ subpopulation. **B** Venn diagram represents mean subpopulation frequencies of ER-α66^+^ ∩ p38 MAPK^+^ (green) and ER-α66^-^ ∩ p38 MAPK^+^ (blue) in TAM. Scatter plots show that p38 MAPK expression is higher in the ER-α66^+^ ∩ p38 MAPK^+^ subpopulation under TAM. **C** Venn diagram represents mean ER-α66^+^ ∩ pS6^+^ subpopulation frequencies. Scatter plots show TAM effects on the ER-α66^+^ ∩ pS6^+^ subpopulations. **D** Venn diagram represents mean ER-α46^+^ ∩ pS6^+^ subpopulation frequencies. Scatter plots show the TAM effects on the ER-α46^+^ ∩ pS6^+^ subpopulation. **E** pS6 mitigates TAM repression on ER-α66. Venn diagram represents mean subpopulation frequencies of ER-α66^+^ ∩ pS6^+^ (green) and ER-α66^+^ ∩ pS6^-^ (blue) in TAM. Scatter plots show TAM inhibition is reduced in the ER-α46^+^ ∩ pS6^+^ subpopulation. **F** Venn diagram represents mean subpopulation frequencies of ER-α46^+^ ∩ pS6^+^ (green) and ER-α46^+^ ∩ pS6^-^ (blue) in TAM. Scatter plot compares TAM effect on ER-α46 between the ER-α46^+^ ∩ pS6^+^ and ER-α66^+^ ∩ pS6^-^ subpopulations. **G** Diagram illustrates ER-α66 regulating downstream p38 MAPK. Diagram also shows pS6 as an upstream ER-α protein target upregulating both ER-α66 and ER-α46. Red lines in scatter plots denote mean values. Detailed statistical tests are reported in **S3, S4 Tables in S1 File** (*p < 0.05, ***p < 0.0001, ns: p > 0.05).

In addition to the PI3K/AKT/mTOR pathway, we sought to scrutinize the interaction between ER isoforms and p38 MAPK in hormone-sensitive BCa cells (MCF-7). Given the significant changes in correlation between p38 MAPK and ER-α66 with and without TAM treatment (⍴ = -0.27 in Neg to ⍴ = 0.83 in TAM, **Fig 5A)**, we hypothesized that TAM affects the p38 MAPK expression level in an ER-α66 dependent manner. We did not observe significant changes in p38 MAPK expression at the population level (**S2 Table in S1 File**), but we did observe that TAM increased the mean p38 MAPK expression level by 17% in the ER-α66^+^ ∩ p38 MAPK^+^ subpopulation (**Fig 7A, S3 Table in S1 File)**. The upregulation of p38 MAPK is associated with the presence of the ER-α66 protein: mean p38 MAPK expression is 31% higher in the ER-α66^+^ ∩ p38 MAPK^+^ subpopulation, as compared to the ER-α66^-^ ∩ p38 MAPK^+^ subpopulation (**Fig 7B, S4 Table in S1 File)**. TAM significantly decreased the mean ER-α66 expression level (77% decrease, **Fig 7A, S3 Table in S1 File)**. The subpopulation analysis suggests a unidirectional relationship between ER-α66 and p38 MAPK, in which TAM affects ER-α66 to alter p38 MAPK pathway (**Fig 7G)**.

### pS6 upregulates ER-α isoforms via the non-canonical ER signaling pathway

Next, we sought to scrutinize the interaction of pS6 (an indicator of activity in the PI3K/pAKT/mTOR signaling pathway) with ER-α isoforms in the hormone-sensitive MCF-7 cells [59]. Unlike pAKT, we did not observe perturbation of pS6 expression in either the whole population (**S6 Fig, S2 Table in S1 File**), the ER-α66^+^ subpopulation (**Fig 7C**), or the ER-α46^+^ subpopulations (**Fig 7D, S3 Table in S1 File)** upon TAM treatment. Instead, TAM affected ER-α isoforms in the pS6^+^ subpopulations. TAM significantly altered the mean ER-α66 expression level in the ER-α66^+^ ∩ pS6^+^ subpopulation and the mean ER-α46 expression level in the ER-α46^+^ ∩ pS6^+^ subpopulation (**Fig 7C and 7D, S3 Table in S1 File)**. In order to understand if ER-α isoform responses are linked to the presence of pS6, we compared the pS6^+^ and the pS6^-^ subpopulations **(Fig 7E and 7F)**. Interestingly, we discovered that pS6 appears to mitigate TAM repression of ER-α66: the mean ER-α66 expression in the pS6^+^ subpopulation is 50% greater than the ER-α66 expression in the pS6^-^ subpopulation **(Fig 7E, S4 Table in S1 File)**. Similarly, we observed that the mean ER-α46 expression is greater in the pS6^+^ subpopulation **(Fig 7F, S4 Table in S1 File)**. Taken together, the single-cell protein analysis suggests that pS6 upregulates both ER-α66 and ER-α46 **(Fig 7G)**.

### pAKT is associated with ER-α46 upon TAM treatment in MDA-MB-231

**Fig 8:**
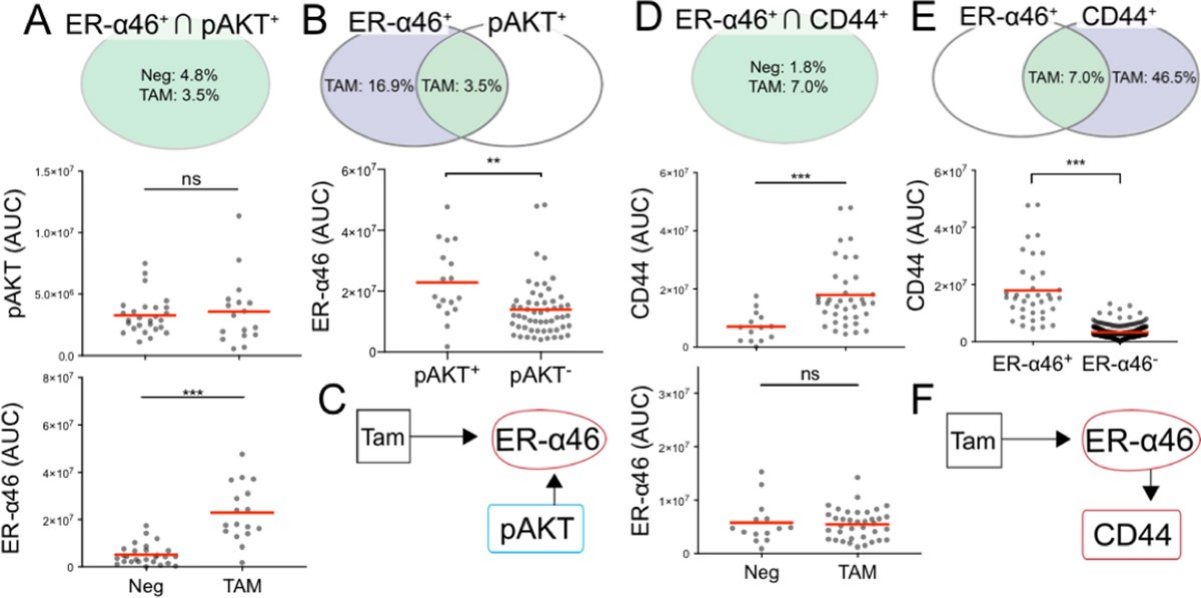
Subpopulation analysis identifies non-canonical signaling interaction between pAKT/CD44 and ER-α46 in MDA-MB-231 cells. **A** Venn diagram represents mean ER-α46^+^ ∩ pAKT^+^ subpopulation frequencies. Scatter plots show TAM effects on the ER-α46^+^ ∩ pAKT^+^ subpopulation. **B** Venn diagram represents mean subpopulation frequencies of ER-α46^+^ ∩ pAKT^+^ (green) and ER-α46^+^ ∩ pAKT^-^ (blue) in TAM. Scatter plot compares the effects of TAM on ER-α46 between the ER-α46^+^ ∩ pAKT^+^ and ER-α46^+^ ∩ pAKT^-^ subpopulations. **C** Diagram illustrates pAKT upregulating ER-α46 in TAM. **D** Venn diagram represents mean ER-α46^+^ ∩ CD44^+^ subpopulation frequencies. Scatter plots show TAM effects on the ER-α46^+^ ∩ CD44^+^ subpopulation. **E** Venn diagram represents mean subpopulation frequencies of ER-α46^+^ ∩ CD44^+^ (green) and ER-α46^+^ ∩ CD44^-^ (blue) in TAM. Scatter plots show that CD44 expression is higher in the ER-α46^+^ ∩ CD44^+^ subpopulation under TAM. **F** Diagram illustrates ER-α46 upregulating downstream CD44. Red lines in scatter plots denote mean values. Detailed statistical tests are reported in **S3, S4 Tables in S1 File** (**p < 0.01, ***p < 0.0001).

In hormone-insensitive cancer, TAM induces apoptosis by inhibiting pAKT in a dose independent pathway [60]. We examined whether TAM modulates the expression level of pAKT via the ER-α46 associated non-canonical ER signaling pathway **(Fig 8)**. In contrast to MCF-7 cells, we observed no change in the mean expression of pAKT with or without TAM treatment. The observation was the same in both the ER-α46^+^ ∩ pAKT^+^ subpopulation and the overall population **(Fig 8A, S6 Fig, S2 Table in S1 File**). Interestingly, we observed that the presence of pAKT leads to a greater mean ER-α46 expression level under TAM (**Fig 8B, S4 Table in S1 File)**. Spearman’s correlation suggested median correlation between ER-α46 and pAKT (⍴ = 0.61, **Fig 5B**). Accordingly, we suspect a strong interaction between ER-α46 and pAKT in hormone-insensitive MDA-MB-231 cells upon TAM treatment (**Fig 8C**).

### CD44 is the downstream protein target in the non-canonical ER signaling pathway

We next sought to scrutinize the non-canonical ER signaling pathway in the hormone-insensitive MDA-MB-231 cells. While we did not observe mean CD44 expression level changes upon TAM treatment at the population level **(S2 Table in S1 File)**, we did observe that TAM increases the mean CD44 expression level by 60% in the ER-α46^+^ population (**Fig 8D, S3 Table in S1 File**). Further, the mean CD44 expression level in the ER-α46^+^ ∩ CD44^+^ subpopulation is 53% greater than the mean CD44 expression level in the ER-α46^-^ ∩ CD44^+^ subpopulation (**Fig 8E**, **S4 Table in S1 File**). On the other hand, the CD44^+^ subpopulation did not see ER-α46 influenced by TAM (**Fig 8D, S3 Table in S1 File**). Taken together, ER-α46 induces CD44, while CD44 does not appear to regulate ER-α46 (**Fig 8F)**.

### Principal component analysis suggests dominant ER signaling targets in BCa cell lines

**Fig 9:**
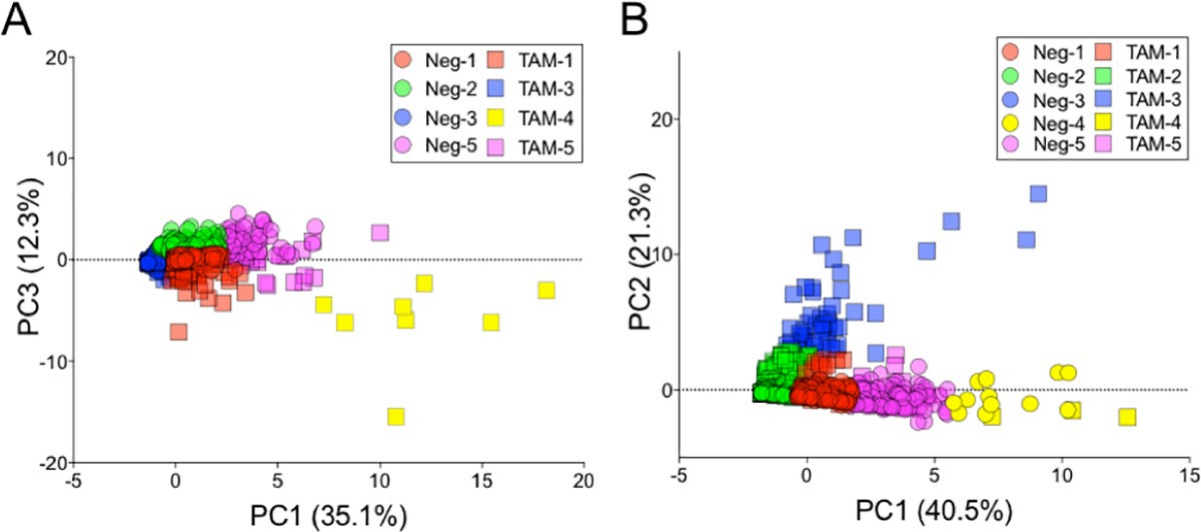
ER signaling pathway subpopulations and putative protein targets are indicated using PCA with K-means clustering of MCF-7 and MDA-MB-231 cells. PCA score plots of A TAM-treated and non-treated MCF-7 and B MDA-MB-231 classify different clusters based on the first three PC’s. TAM-treated (squares) and non-treated (circles) datasets are combined. Colors denote different clusters. Number of clusters is optimized using the elbow method (S6 Fig in S1 File) [61].

After detecting interactions between the ER-α isoforms and ER signaling targets, we applied PCA with K-means clustering to distinguish the BCa subpopulations responding to TAM. First, we pooled the non-treated and TAM-treated MCF-7 datasets and performed PC scoring (using linear combinations of ER signaling markers, **Fig 9A, S6 Fig in S1 File**). We found that PC1 separates the cluster Neg-2 (green) from the cluster TAM-4 (yellow; **Fig 9A**). Interestingly, 100% of clusters 2 and 4 consisted of non-treated and TAM-treated MCF-7 cells, respectively. Based on high correlations with PC1, we conclude that cJUN (⍴_cJUN_ = 0.45), pAKT (⍴_pAKT_ = 0.45), and pS6 (⍴_pS6_ = 0.40) are dominant factors for differentiating between non-treated and TAM-treated subpopulations–indicating that the clusters Neg-2 and TAM-4 differentiate from the rest of the cells owing to the non-canonical ER signaling (**S7 Fig in S1 File)**. PC1 affects 35.1% and PC3 contributes 11.2% of total variance. PC3 separates the non-treated cells in the clusters Neg-2 (green) and 5 (Neg, TAM; magenta) from the rest. Likewise, PC3 correlates most strongly with ER-α46 (⍴_ER-α46_ = -0.48), Cyclin A (⍴_ER-α46_ = -0.48), ER-α66 (⍴_ER-α46_ = 0.64; **Fig 9A, S6 Fig in S1 File**).

Next, we sought to which, if any, dominant ER signaling protein targets are distinct to subpopulations of hormone-insensitive MDA-MB-231 cells. PCA was performed with the mixed dataset of non-treated and TAM-treated cells to elucidate subpopulation phenotypes (**Fig 9B**). The dataset variability is described largely by PC1 (40.5%) and PC2 (21.3%; **S7 Fig in S1 File**). PC1 partitions all the clusters except the cluster 3 (blue; **Fig 9B**). The PC1 partitioning is strongly associated with cJUN (⍴_cJUN_ = 0.43), p38 MAPK (⍴_p38MAPK_ = 0.47), pAKT (⍴_pAKT_ = 0.47), pS6 (⍴_pS6_ = 0.42; **S7 Fig in S1 File**). PC2 separates the non-treated (circle) and TAM-treated (square) datasets (**Fig 9B**). The factors strongly associated with PC2 are ER-α46 (⍴_ER-α46_ = 0.64) and Cyclin A (⍴_CyclinA_ = 0.67; **S7 Fig in S1 File**).

## Discussion

Here, single-cell immunoblotting reports ER-α isoform heterogeneity in both hormone-sensitive and hormone-insensitive BCa. We classify and compare ER-α isoform expression among cell subpopulations in cell lines to understand the BCa cell lines as ER-signaling models. With PCA explaining 48.6% of the variance of 7 ER-signaling targets, we find similar ER signaling expression levels between the cell lines and the tissue specimens (**Fig 2B**) [62, 63].

In bulk assays, stimulation of BCa cells with TAM is known to modulate ER-α66 mRNA [51, 53, 64]. By single-cell immunoblot, we observed a decrease in ER-α66 protein expression after TAM treatment (**Fig 3D**). While TAM is understood to stabilize ER-α66 protein expression [65, 66], these studies use different tamoxifen metabolites (i.e. 4-hydroxytamoxifen), and concentrations (i.e., < 1 μM). In contrast, TAM at > 1 μM was reported to degrade ER-α66 by the proteasome [67]. The effect of 1 > μM non-metabolized TAM on ER-α66 protein expression has not been investigated previously. Measuring ER-α isoforms at a single cell level with various concentrations of ER modulators from derived and different ER model cell lines would provide more insight on pharmacological research.

In hormone-sensitive MCF-7, the ER-α66 protein expression level is lower in the same cells expressing ER-α46 (**Fig 3C)**, mirroring with the repressive estrogenic activity of ER-α66 by ER-α46 at the transcription level as reported [15, 17]. However, the positive correlation between ER-α66 and ER-α46 under no treatment and the loss of the correlation under TAM suggest that ER-α46 may not directly inhibit ER-α66 (**Fig 5A)**. Indeed, in the MCF-7 subpopulation expressing both ER-α66 and ER-α46, we did not observe a further decrease of mean ER-α66 expression under TAM (**Fig 3F**).

Although a few studies have reported that ER-α46 inhibits cell growth in the presence of the ER modulator (TAM/E2) [27, 42], the TAM effect on ER-α46 expression has not been investigated in BCa models. The truncated ER-α46 possesses the ligand binding domain that interacts with TAM/E2 [68]. In human macrophages, the E2 treatment increases ER-α46 transcription by inducing the promoter F of the ER-α gene (*ESR1*) [54]. Thus, one possible mechanism is a change in promoter activity in which E2 or TAM mediates alternative splicing to generate ER-α46 [42, 54]. Although *ESR1* recruitment in a promoter F region is known to increase the ER-α46 expression level in the MCF-7 cells [69], the TAM regulation of the *ESR1* promoter activity is unknown. Our data indicate that TAM/E2 treatment increases ER-α46 expression level in the MCF-7 cells (**Fig 3D and 3F, S5A Fig in S1 File**). Because the TAM effect in the ligand-dependent AF-1 domain varies with specific cell and promoter types, further examination of transcription and translation is needed to understand TAM mechanisms at the level of individual cells.

Single-cell multiplexing of ER signaling provides detailed examination of cell-to-cell variation in canonical and non-canonical ER-α actions (**Fig 1A**). Looking at subpopulations expressing specific ER signaling proteins, pair-wise comparison of ER-α isoforms and ER signaling proteins shows TAM enhancing the correlation between ER-α isoforms and ER signaling proteins (**Fig 5**). Strong ER-α isoform correlations with pS6, CD44, and Cyclin A in TAM treated cells implicates the non-canonical ER signaling pathway (**Fig 5**).

Importantly, ER-α isoforms appear to associate with the PI3K/AKT/mTOR pathway, which regulates BCa cell survival and proliferation [70–72]. By examining both pAKT^+^ and pS6^+^ in the MCF-7 subpopulations, pAKT and pS6 arise as potentially mitigating TAM repression of ER-α66 and induction of ER-α46 (**Figs 6C, 6E, 7F and 7G**). As confirmed by a recent clinical ER^+^ BCa data analysis [73], this subpopulation can contribute to TAM resistance. Reciprocally, the subpopulation expressing both of the ER-α isoforms (MCF Cell Type 1) lessens the TAM effect on pAKT (**Fig 6F)**. This relationship between pAKT and ER-α isoforms resembles the reduced TAM efficacy observed in tumor tissues co-expressing ER-α66 and pAKT, by IHC [74]. Combining inhibitors targeting the mTOR pathway with anti-estrogen treatments may create synergetic therapeutic effects for ER-α46^+^ ∩ pS6^+^ and ER-α46^+^ ∩ pAKT^+^ harboring BCa patients [75, 76]. Indeed, the combination of everolimus (mTOR inhibitor) with aromatase inhibitors has shown to increase progression-free survival for patients with advanced ER-α^+^/HER2^-^ BCa [77, 78].

Lacking ER-α66 isoform expression, the hormone-insensitive MDA-MB-231 cells exhibited a rare subpopulation (4.2% frequency) which had an ER-α46 expression level (MDA Cell Type 1) that was higher than the hormone-sensitive MCF-7 subpopulation (5.5% frequency) with ER-α46^+^ (MCF Cell Type 1) (**Figs 3B, 4B and 4C)**. Although TAM does not affect the ER-α46 expression level in the MDA-MB-231 subpopulation, the frequency of MDA-MB-231 cells expressing ER-α46 is increased from 4.2% to 20.4% (**Fig 4D and 4E)**. Similar to the hormone-sensitive MCF-7 cells, the subpopulation of cells with pAKT^+^ sees an increase in the ER-α46 expression level after TAM treatment (**Fig 8A–8C**). The combinatorial treatment of PIP5K1α/pAKT inhibitor and TAM may enhance the sensitivity to hormone therapy on this pAKT^+^ ∩ ER-α46^+^ MDA-MB-231 subpopulation [79].

By PCA, we observed pAKT and pS6 as dominant factors in demarcating the hormone-sensitive MCF-7 and hormone-insensitive MDA-MB-231 subpopulations (**Figs 6, 7 and 9)**. pAKT and pS6 are specific to the MCF-7 subpopulations under TAM treatment (yellow, **Fig 9A**). Indeed, pAKT appears to induce ER-α isoforms in these MCF-7 and MDA-MB-231 subpopulations (**Figs 6 and 8)**. For MDA-MB-231, the PCA suggests that ER-α46 is a biomarker specific to the subpopulations responsive to TAM (**Fig 9B)**.

With a focus on validation and application of precision single-cell protein measurement tools, we scrutinize unmodified, endogenous protein isoforms in signaling pathways using two model BCa cell lines. We verify the isoform selectivity, analytical sensitivity, throughput, and monitoring of response to drug treatment. Looking forward, the integration of single-cell immunoblotting and gene knockout/overexpression of other BCa cell lines would offer a deep dive into the signaling cascades [49, 80, 81]. Further, subcellular analysis of ER isoforms and signaling proteins would tease apart the role of membrane and nuclear protein forms to boost understanding of membrane-bound ER-α [16, 17, 82]. Given the importance of truncated oncoprotein isoforms in the development of drug resistance and as potential therapeutic targets, high-selectivity and multiplexed cytometry tools–such as that described here–are a critical component for advancing personalized therapies to benefit each individual patient.

## Supporting information

S1 File (PPTX)

S1 Raw images (PDF)

S1 Text (DOCX)
