# Supplementary material for: Single-cell immunoblotting resolves estrogen receptor-α isoforms in breast cancer
Source: PLoS One. 2021 Jul 27;16(7):e0254783. doi: 10.1371/journal.pone.0254783 (PMC8315538; doi:10.1371/journal.pone.0254783)
Supplement: S1 File — (PPTX) [file pone.0254783.s001.pptx]

## Slide 1
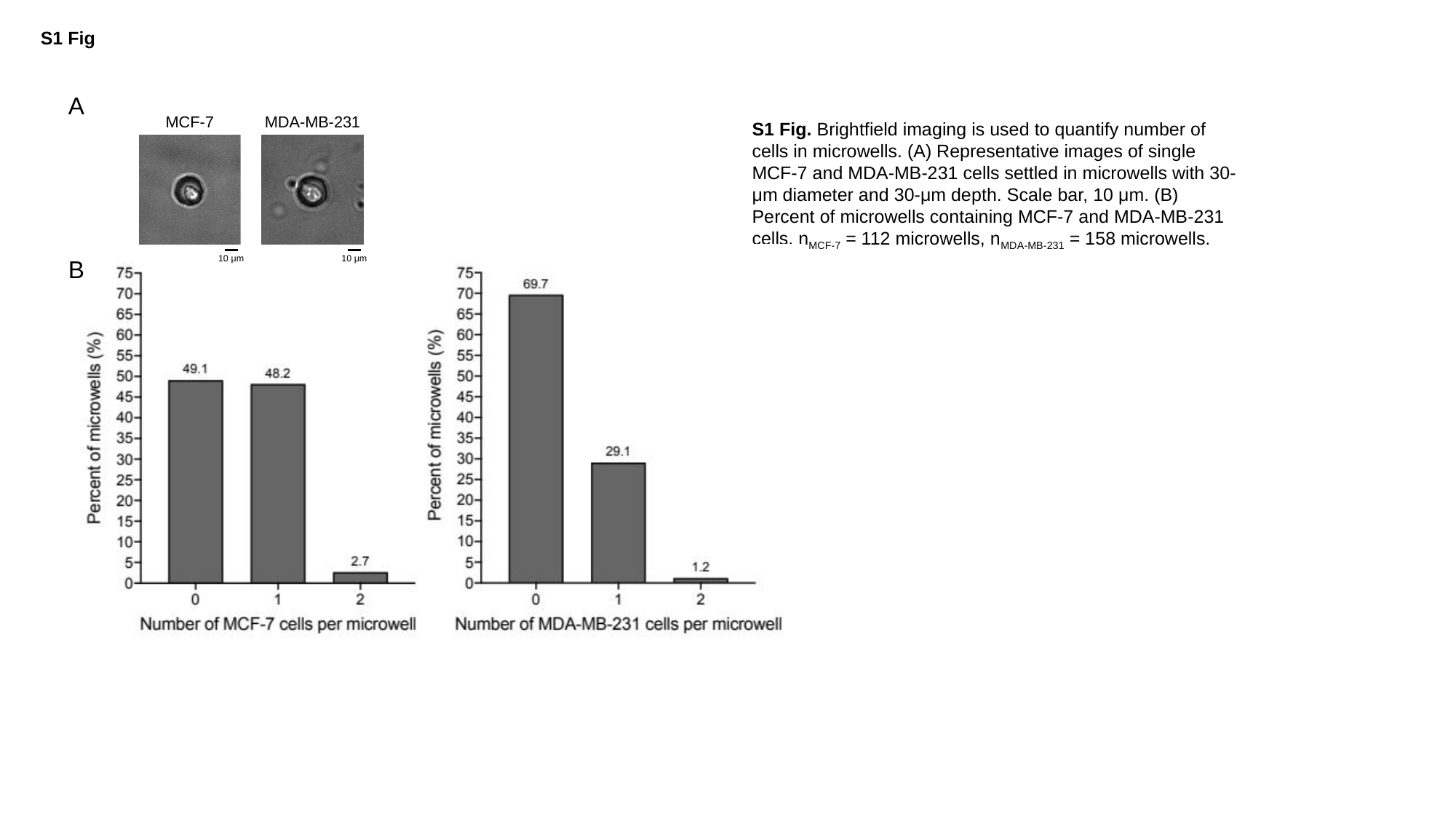

S1 Fig
A
MCF-7
MDA-MB-231
S1 Fig. Brightfield imaging is used to quantify number of cells in microwells. (A) Representative images of single MCF-7 and MDA-MB-231 cells settled in microwells with 30-μm diameter and 30-μm depth. Scale bar, 10 μm. (B) Percent of microwells containing MCF-7 and MDA-MB-231 cells. nMCF-7 = 112 microwells, nMDA-MB-231 = 158 microwells.
10 μm
10 μm
B

## Slide 2
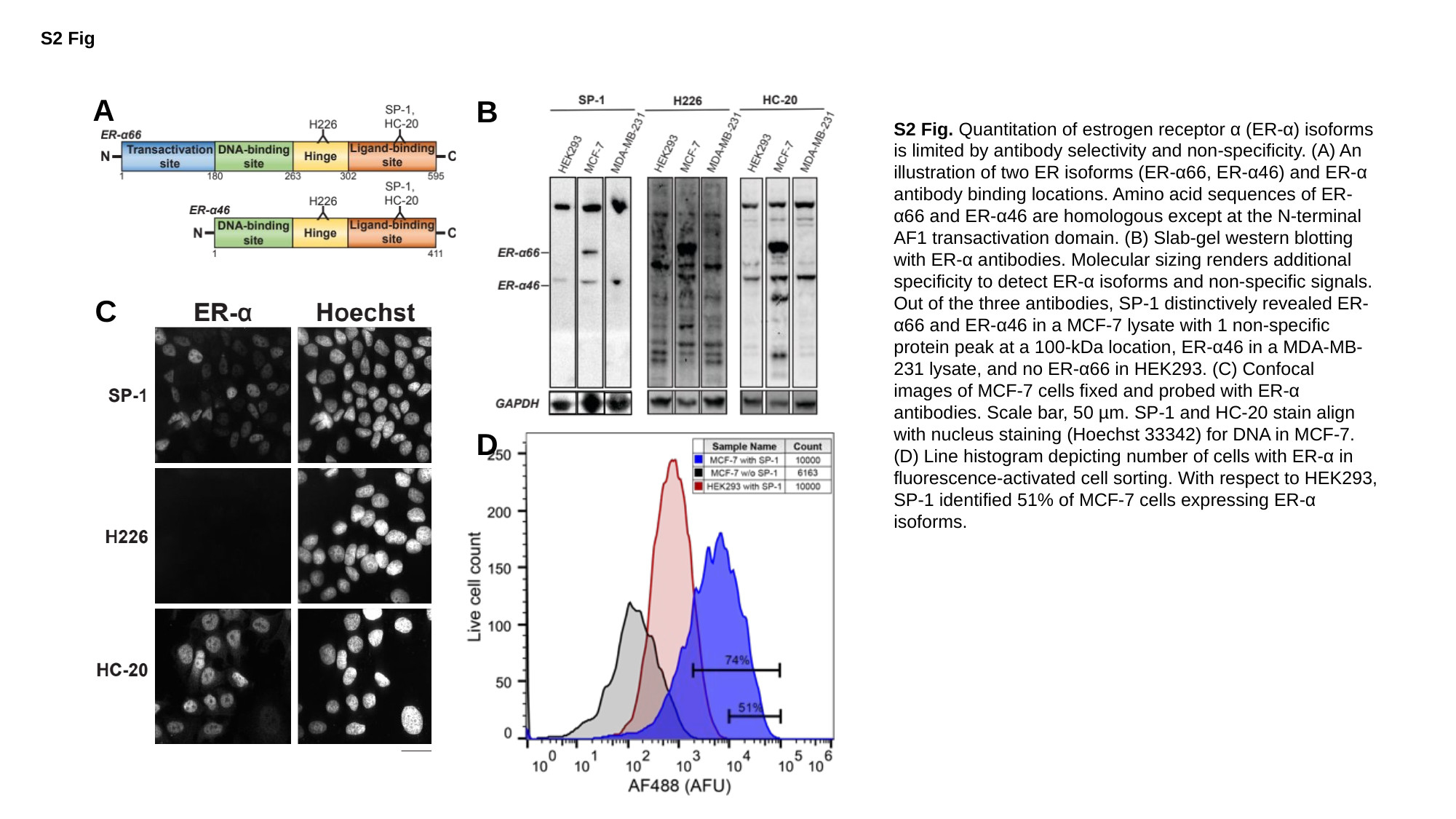

S2 Fig
B
A
C
D
S2 Fig. Quantitation of estrogen receptor α (ER-α) isoforms is limited by antibody selectivity and non-specificity. (A) An illustration of two ER isoforms (ER-α66, ER-α46) and ER-α antibody binding locations. Amino acid sequences of ER-α66 and ER-α46 are homologous except at the N-terminal AF1 transactivation domain. (B) Slab-gel western blotting with ER-α antibodies. Molecular sizing renders additional specificity to detect ER-α isoforms and non-specific signals. Out of the three antibodies, SP-1 distinctively revealed ER-α66 and ER-α46 in a MCF-7 lysate with 1 non-specific protein peak at a 100-kDa location, ER-α46 in a MDA-MB-231 lysate, and no ER-α66 in HEK293. (C) Confocal images of MCF-7 cells fixed and probed with ER-α antibodies. Scale bar, 50 µm. SP-1 and HC-20 stain align with nucleus staining (Hoechst 33342) for DNA in MCF-7. (D) Line histogram depicting number of cells with ER-α in fluorescence-activated cell sorting. With respect to HEK293, SP-1 identified 51% of MCF-7 cells expressing ER-α isoforms.

## Slide 3
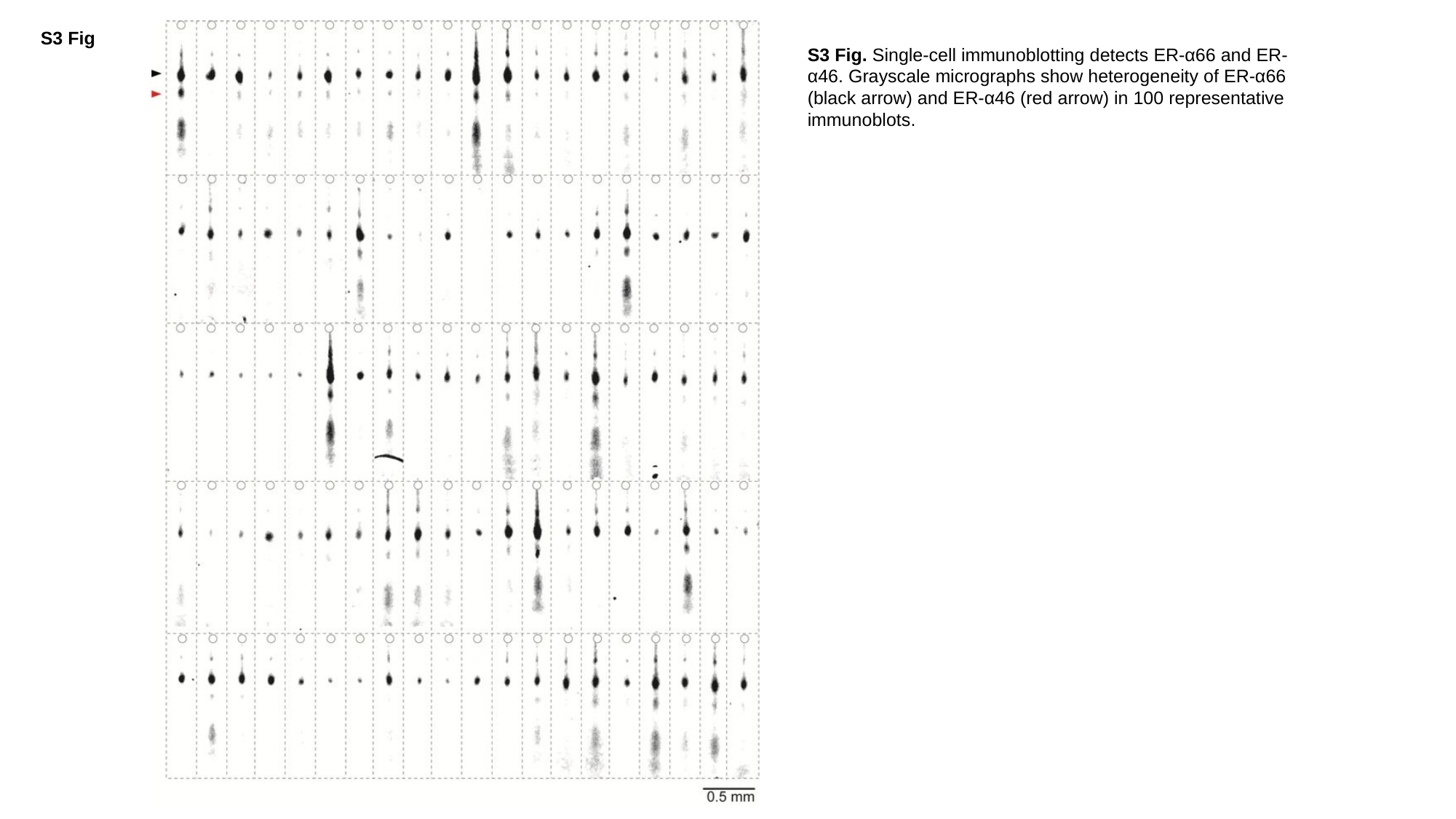

S3 Fig
S3 Fig. Single-cell immunoblotting detects ER-α66 and ER-α46. Grayscale micrographs show heterogeneity of ER-α66 (black arrow) and ER-α46 (red arrow) in 100 representative immunoblots.

## Slide 4
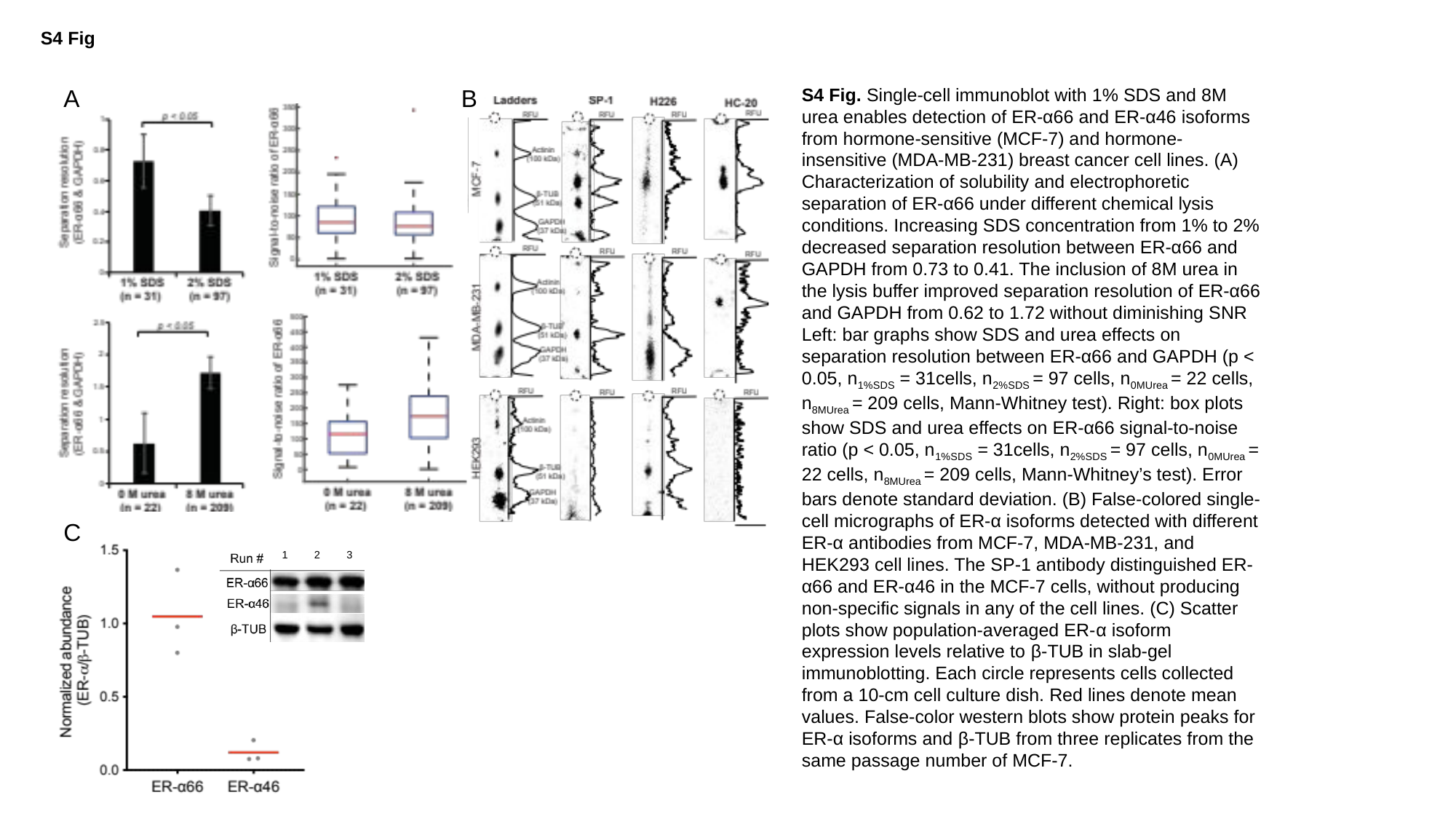

S4 Fig
A
B
S4 Fig. Single-cell immunoblot with 1% SDS and 8M urea enables detection of ER-α66 and ER-α46 isoforms from hormone-sensitive (MCF-7) and hormone-insensitive (MDA-MB-231) breast cancer cell lines. (A) Characterization of solubility and electrophoretic separation of ER-α66 under different chemical lysis conditions. Increasing SDS concentration from 1% to 2% decreased separation resolution between ER-α66 and GAPDH from 0.73 to 0.41. The inclusion of 8M urea in the lysis buffer improved separation resolution of ER-α66 and GAPDH from 0.62 to 1.72 without diminishing SNR Left: bar graphs show SDS and urea effects on separation resolution between ER-α66 and GAPDH (p < 0.05, n1%SDS = 31cells, n2%SDS = 97 cells, n0MUrea = 22 cells, n8MUrea = 209 cells, Mann-Whitney test). Right: box plots show SDS and urea effects on ER-α66 signal-to-noise ratio (p < 0.05, n1%SDS = 31cells, n2%SDS = 97 cells, n0MUrea = 22 cells, n8MUrea = 209 cells, Mann-Whitney’s test). Error bars denote standard deviation. (B) False-colored single-cell micrographs of ER-α isoforms detected with different ER-α antibodies from MCF-7, MDA-MB-231, and HEK293 cell lines. The SP-1 antibody distinguished ER-α66 and ER-α46 in the MCF-7 cells, without producing non-specific signals in any of the cell lines. (C) Scatter plots show population-averaged ER-α isoform expression levels relative to β-TUB in slab-gel immunoblotting. Each circle represents cells collected from a 10-cm cell culture dish. Red lines denote mean values. False-color western blots show protein peaks for ER-α isoforms and β-TUB from three replicates from the same passage number of MCF-7.
C
1
2
3

## Slide 5
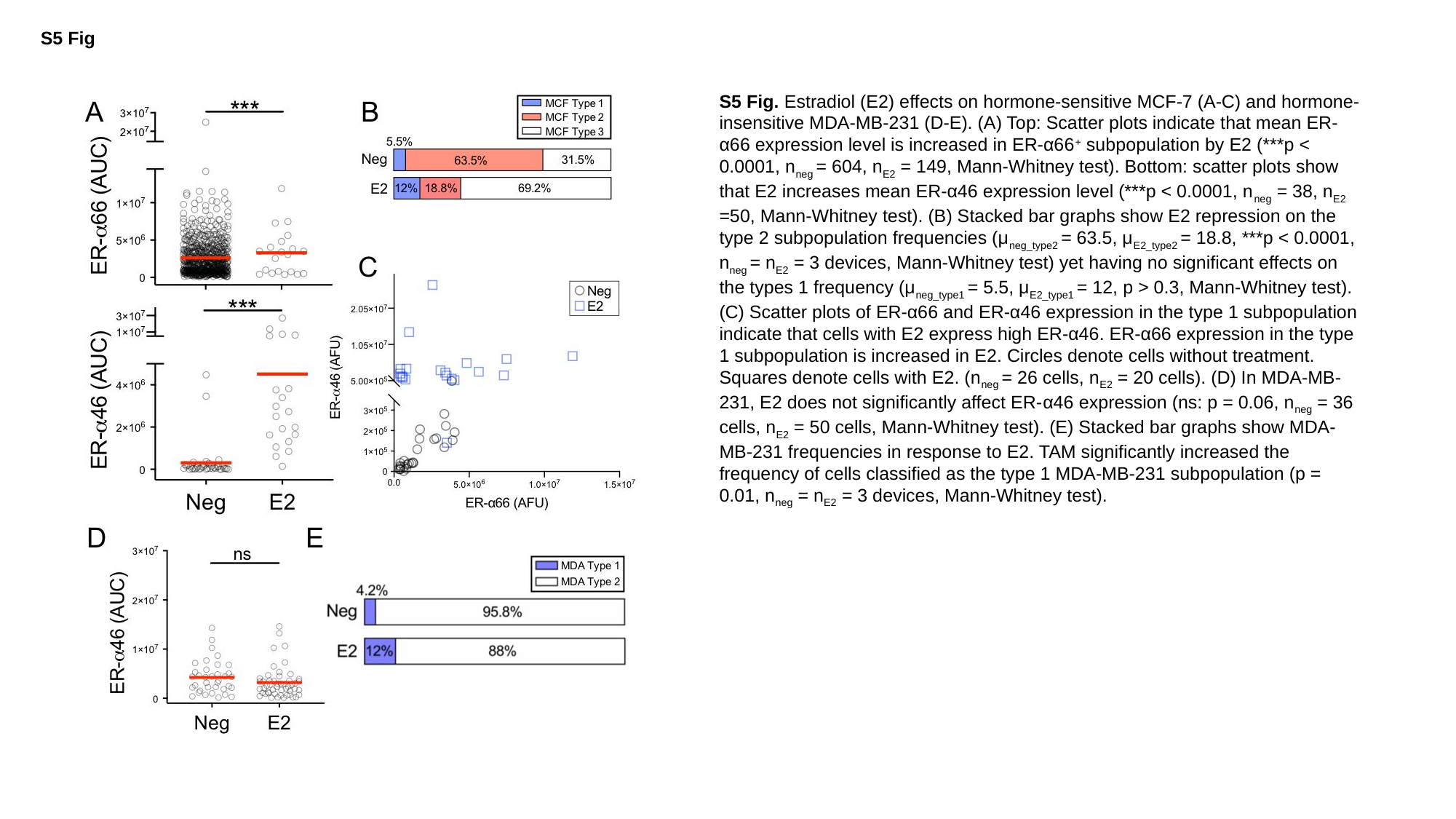

S5 Fig
S5 Fig. Estradiol (E2) effects on hormone-sensitive MCF-7 (A-C) and hormone-insensitive MDA-MB-231 (D-E). (A) Top: Scatter plots indicate that mean ER-α66 expression level is increased in ER-α66+ subpopulation by E2 (***p < 0.0001, nneg = 604, nE2 = 149, Mann-Whitney test). Bottom: scatter plots show that E2 increases mean ER-α46 expression level (***p < 0.0001, nneg = 38, nE2 =50, Mann-Whitney test). (B) Stacked bar graphs show E2 repression on the type 2 subpopulation frequencies (μneg_type2 = 63.5, μE2_type2 = 18.8, ***p < 0.0001, nneg = nE2 = 3 devices, Mann-Whitney test) yet having no significant effects on the types 1 frequency (μneg_type1 = 5.5, μE2_type1 = 12, p > 0.3, Mann-Whitney test). (C) Scatter plots of ER-α66 and ER-α46 expression in the type 1 subpopulation indicate that cells with E2 express high ER-α46. ER-α66 expression in the type 1 subpopulation is increased in E2. Circles denote cells without treatment. Squares denote cells with E2. (nneg = 26 cells, nE2 = 20 cells). (D) In MDA-MB-231, E2 does not significantly affect ER-α46 expression (ns: p = 0.06, nneg = 36 cells, nE2 = 50 cells, Mann-Whitney test). (E) Stacked bar graphs show MDA-MB-231 frequencies in response to E2. TAM significantly increased the frequency of cells classified as the type 1 MDA-MB-231 subpopulation (p = 0.01, nneg = nE2 = 3 devices, Mann-Whitney test).

## Slide 6
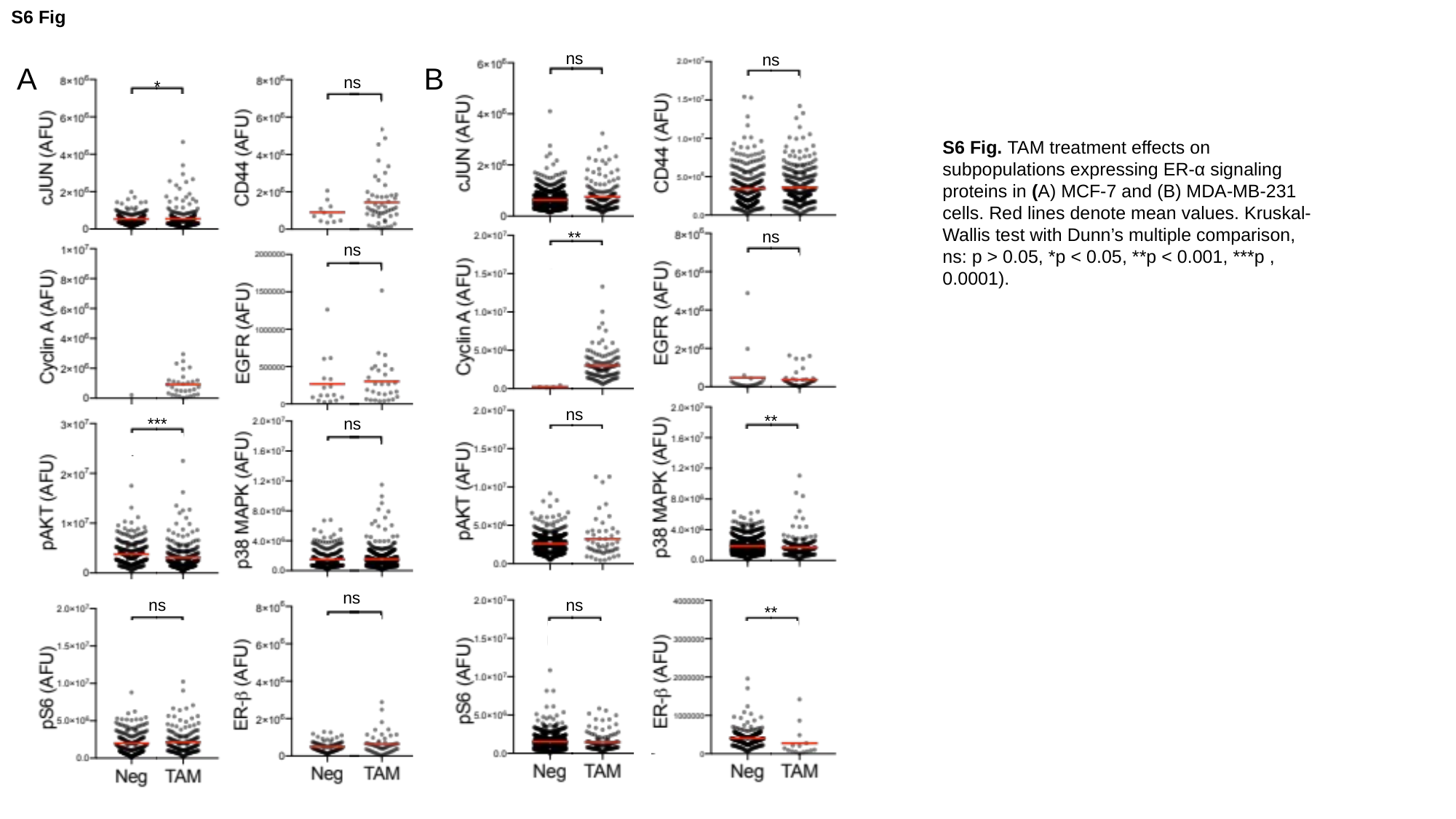

S6 Fig
ns
ns
A
B
ns
*
S6 Fig. TAM treatment effects on subpopulations expressing ER-α signaling proteins in (A) MCF-7 and (B) MDA-MB-231 cells. Red lines denote mean values. Kruskal-Wallis test with Dunn’s multiple comparison, ns: p > 0.05, *p < 0.05, **p < 0.001, ***p , 0.0001).
ns
**
ns
ns
**
***
ns
ns
ns
ns
**

## Slide 7
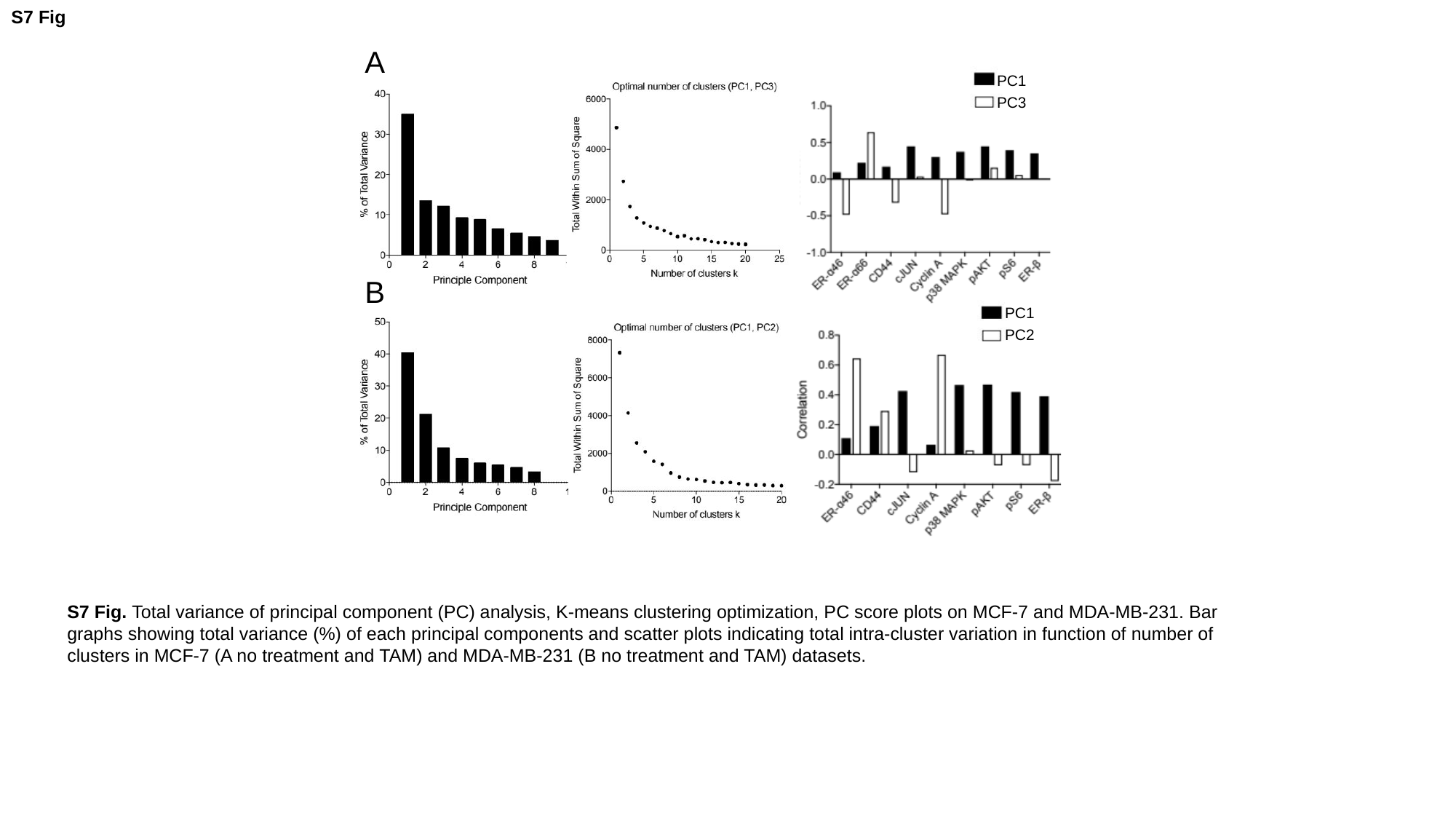

S7 Fig
A
PC1
PC3
B
PC1
PC2
S7 Fig. Total variance of principal component (PC) analysis, K-means clustering optimization, PC score plots on MCF-7 and MDA-MB-231. Bar graphs showing total variance (%) of each principal components and scatter plots indicating total intra-cluster variation in function of number of clusters in MCF-7 (A no treatment and TAM) and MDA-MB-231 (B no treatment and TAM) datasets.

## Slide 8
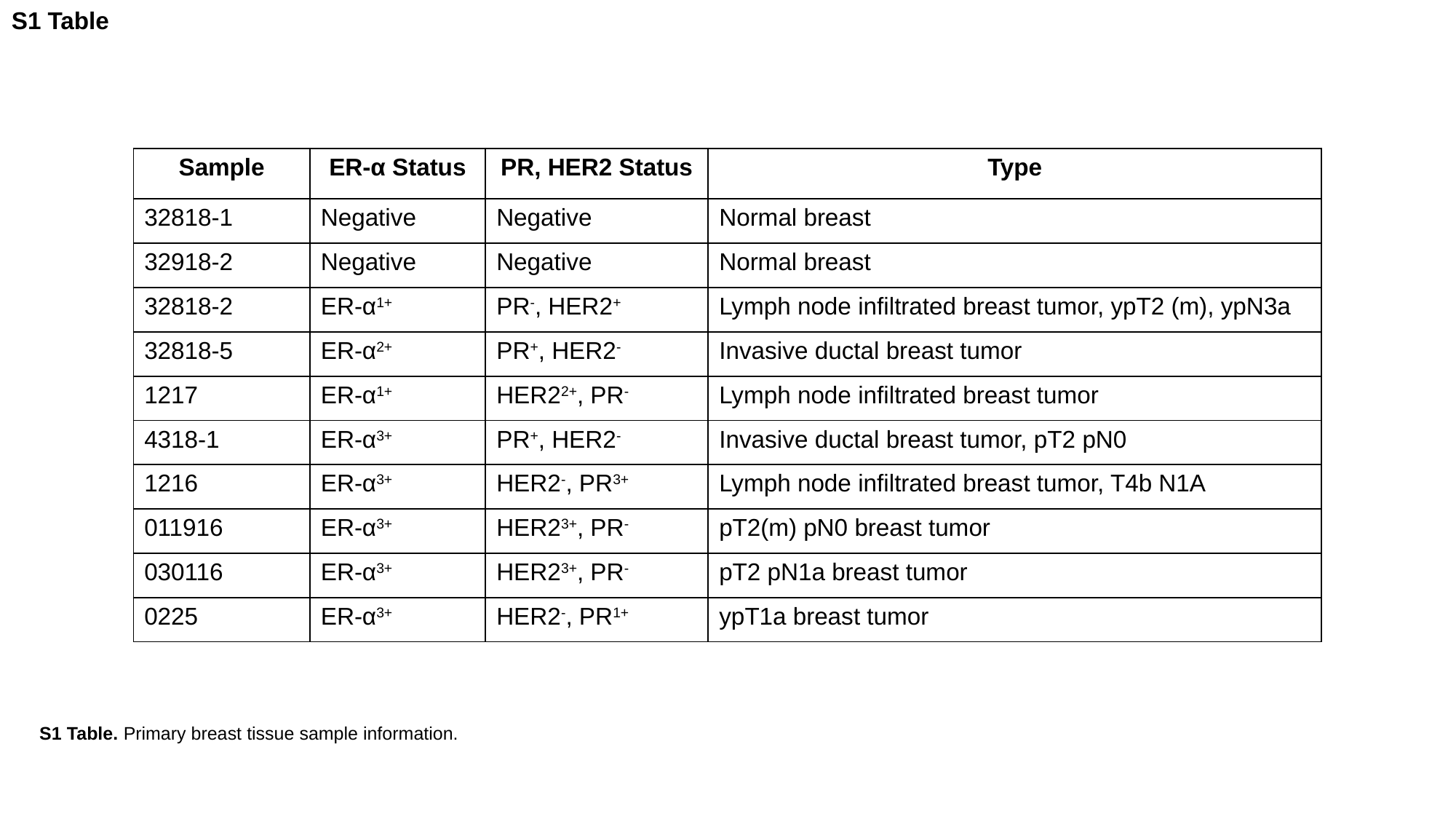

S1 Table
| Sample | ER-α Status | PR, HER2 Status | Type |
| --- | --- | --- | --- |
| 32818-1 | Negative | Negative | Normal breast |
| 32918-2 | Negative | Negative | Normal breast |
| 32818-2 | ER-α1+ | PR-, HER2+ | Lymph node infiltrated breast tumor, ypT2 (m), ypN3a |
| 32818-5 | ER-α2+ | PR+, HER2- | Invasive ductal breast tumor |
| 1217 | ER-α1+ | HER22+, PR- | Lymph node infiltrated breast tumor |
| 4318-1 | ER-α3+ | PR+, HER2- | Invasive ductal breast tumor, pT2 pN0 |
| 1216 | ER-α3+ | HER2-, PR3+ | Lymph node infiltrated breast tumor, T4b N1A |
| 011916 | ER-α3+ | HER23+, PR- | pT2(m) pN0 breast tumor |
| 030116 | ER-α3+ | HER23+, PR- | pT2 pN1a breast tumor |
| 0225 | ER-α3+ | HER2-, PR1+ | ypT1a breast tumor |
S1 Table. Primary breast tissue sample information.

## Slide 9
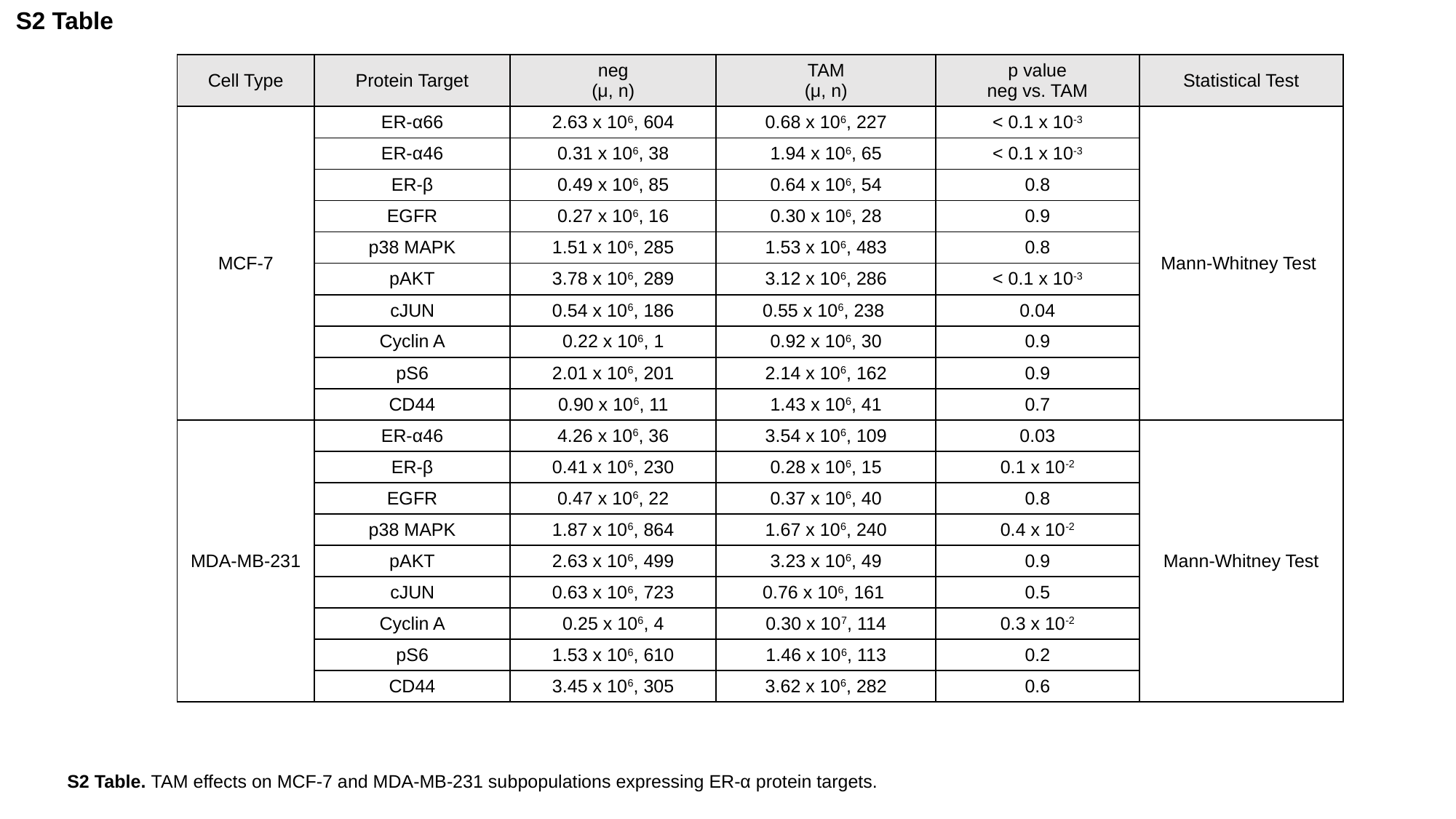

S2 Table
| Cell Type | Protein Target | neg (μ, n) | TAM (μ, n) | p value neg vs. TAM | Statistical Test |
| --- | --- | --- | --- | --- | --- |
| MCF-7 | ER-α66 | 2.63 x 10^6^, 604 | 0.68 x 10^6^, 227 | < 0.1 x 10^-3^ | Mann-Whitney Test |
| | ER-α46 | 0.31 x 10^6^, 38 | 1.94 x 10^6^, 65 | < 0.1 x 10^-3^ | |
| | ER-β | 0.49 x 10^6^, 85 | 0.64 x 10^6^, 54 | 0.8 | |
| | EGFR | 0.27 x 10^6^, 16 | 0.30 x 10^6^, 28 | 0.9 | |
| | p38 MAPK | 1.51 x 10^6^, 285 | 1.53 x 10^6^, 483 | 0.8 | |
| | pAKT | 3.78 x 10^6^, 289 | 3.12 x 10^6^, 286 | < 0.1 x 10^-3^ | |
| | cJUN | 0.54 x 10^6^, 186 | 0.55 x 10^6^, 238 | 0.04 | |
| | Cyclin A | 0.22 x 10^6^, 1 | 0.92 x 10^6^, 30 | 0.9 | |
| | pS6 | 2.01 x 10^6^, 201 | 2.14 x 10^6^, 162 | 0.9 | |
| | CD44 | 0.90 x 10^6^, 11 | 1.43 x 10^6^, 41 | 0.7 | |
| MDA-MB-231 | ER-α46 | 4.26 x 10^6^, 36 | 3.54 x 10^6^, 109 | 0.03 | Mann-Whitney Test |
| | ER-β | 0.41 x 10^6^, 230 | 0.28 x 10^6^, 15 | 0.1 x 10^-2^ | |
| | EGFR | 0.47 x 10^6^, 22 | 0.37 x 10^6^, 40 | 0.8 | |
| | p38 MAPK | 1.87 x 10^6^, 864 | 1.67 x 10^6^, 240 | 0.4 x 10^-2^ | |
| | pAKT | 2.63 x 10^6^, 499 | 3.23 x 10^6^, 49 | 0.9 | |
| | cJUN | 0.63 x 10^6^, 723 | 0.76 x 10^6^, 161 | 0.5 | |
| | Cyclin A | 0.25 x 10^6^, 4 | 0.30 x 10^7^, 114 | 0.3 x 10^-2^ | |
| | pS6 | 1.53 x 10^6^, 610 | 1.46 x 10^6^, 113 | 0.2 | |
| | CD44 | 3.45 x 10^6^, 305 | 3.62 x 10^6^, 282 | 0.6 | |
S2 Table. TAM effects on MCF-7 and MDA-MB-231 subpopulations expressing ER-α protein targets.

## Slide 10
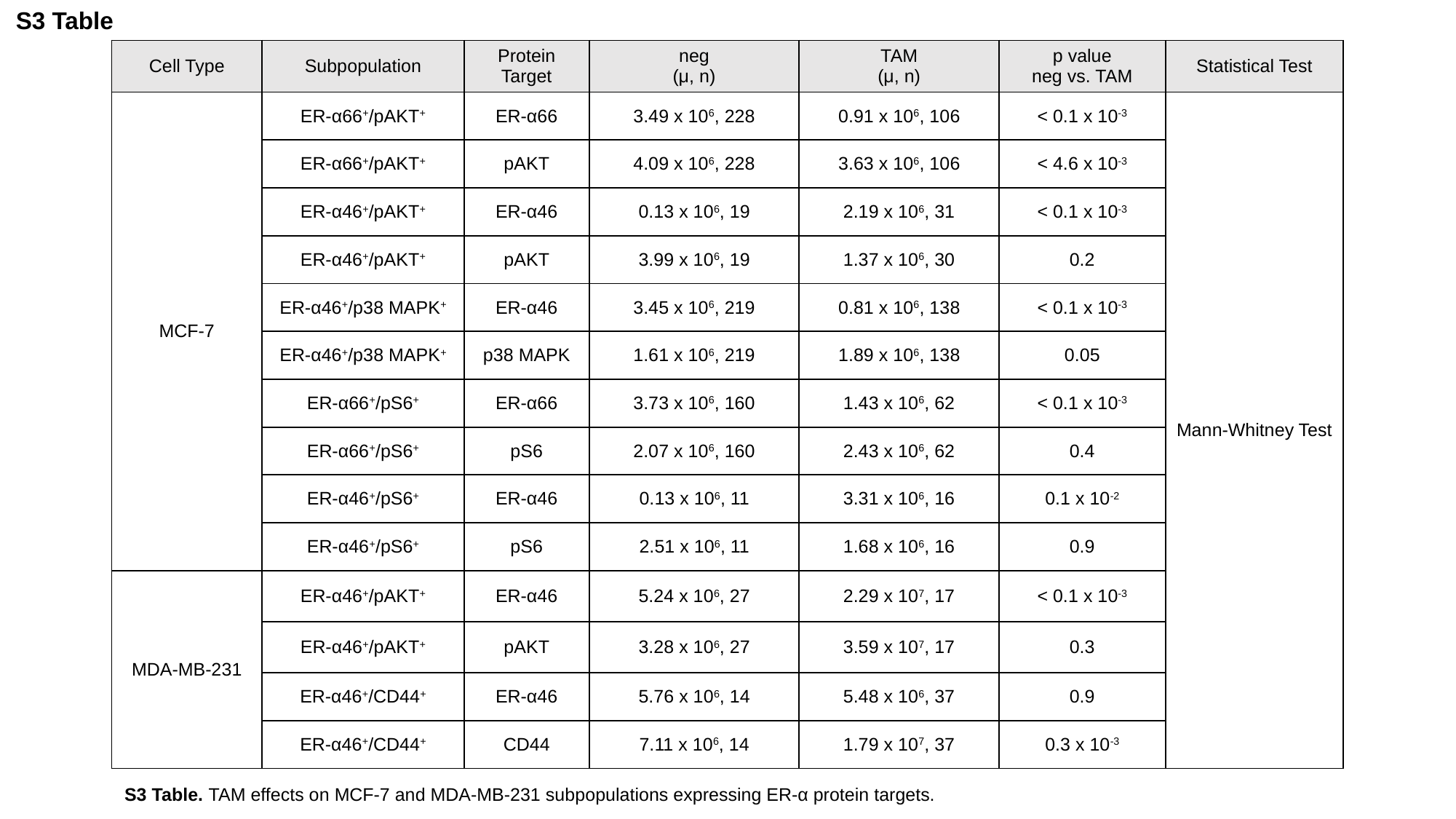

S3 Table
| Cell Type | Subpopulation | Protein Target | neg (μ, n) | TAM (μ, n) | p value neg vs. TAM | Statistical Test |
| --- | --- | --- | --- | --- | --- | --- |
| MCF-7 | ER-α66+/pAKT+ | ER-α66 | 3.49 x 10^6^, 228 | 0.91 x 10^6^, 106 | < 0.1 x 10^-3^ | Mann-Whitney Test |
| | ER-α66+/pAKT+ | pAKT | 4.09 x 10^6^, 228 | 3.63 x 10^6^, 106 | < 4.6 x 10^-3^ | |
| | ER-α46+/pAKT+ | ER-α46 | 0.13 x 10^6^, 19 | 2.19 x 10^6^, 31 | < 0.1 x 10^-3^ | |
| | ER-α46+/pAKT+ | pAKT | 3.99 x 10^6^, 19 | 1.37 x 10^6^, 30 | 0.2 | |
| | ER-α46+/p38 MAPK+ | ER-α46 | 3.45 x 10^6^, 219 | 0.81 x 10^6^, 138 | < 0.1 x 10^-3^ | |
| | ER-α46+/p38 MAPK+ | p38 MAPK | 1.61 x 10^6^, 219 | 1.89 x 10^6^, 138 | 0.05 | |
| | ER-α66+/pS6+ | ER-α66 | 3.73 x 10^6^, 160 | 1.43 x 10^6^, 62 | < 0.1 x 10^-3^ | |
| | ER-α66+/pS6+ | pS6 | 2.07 x 10^6^, 160 | 2.43 x 10^6^, 62 | 0.4 | |
| | ER-α46+/pS6+ | ER-α46 | 0.13 x 10^6^, 11 | 3.31 x 10^6^, 16 | 0.1 x 10^-2^ | |
| | ER-α46+/pS6+ | pS6 | 2.51 x 10^6^, 11 | 1.68 x 10^6^, 16 | 0.9 | |
| MDA-MB-231 | ER-α46+/pAKT+ | ER-α46 | 5.24 x 10^6^, 27 | 2.29 x 10^7^, 17 | < 0.1 x 10^-3^ | |
| | ER-α46+/pAKT+ | pAKT | 3.28 x 10^6^, 27 | 3.59 x 10^7^, 17 | 0.3 | |
| | ER-α46+/CD44+ | ER-α46 | 5.76 x 10^6^, 14 | 5.48 x 10^6^, 37 | 0.9 | |
| | ER-α46+/CD44+ | CD44 | 7.11 x 10^6^, 14 | 1.79 x 10^7^, 37 | 0.3 x 10^-3^ | |
S3 Table. TAM effects on MCF-7 and MDA-MB-231 subpopulations expressing ER-α protein targets.

## Slide 11
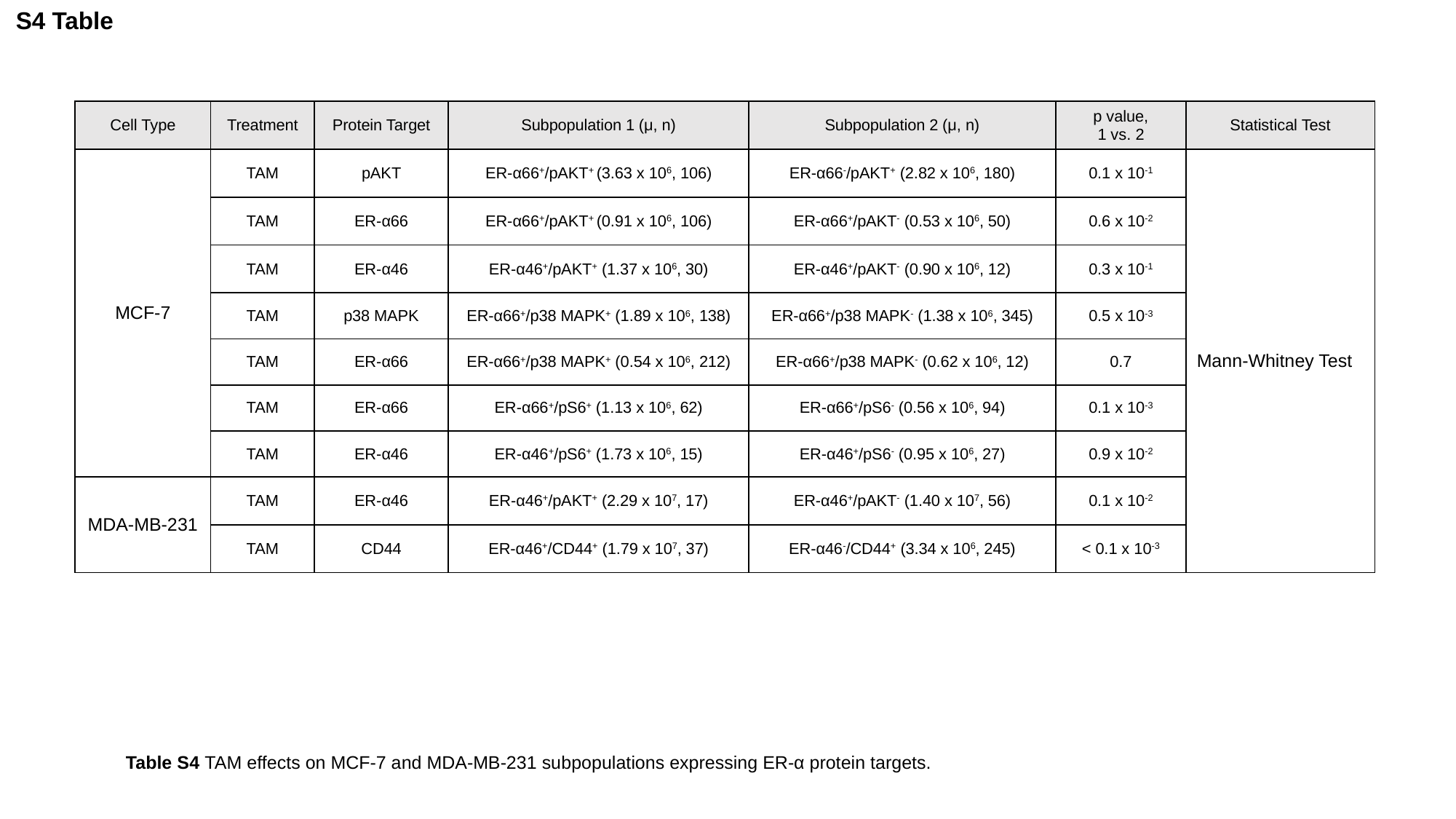

S4 Table
| Cell Type | Treatment | Protein Target | Subpopulation 1 (μ, n) | Subpopulation 2 (μ, n) | p value, 1 vs. 2 | Statistical Test |
| --- | --- | --- | --- | --- | --- | --- |
| MCF-7 | TAM | pAKT | ER-α66+/pAKT+ (3.63 x 10^6^, 106) | ER-α66-/pAKT+ (2.82 x 10^6^, 180) | 0.1 x 10^-1^ | Mann-Whitney Test |
| | TAM | ER-α66 | ER-α66+/pAKT+ (0.91 x 10^6^, 106) | ER-α66+/pAKT- (0.53 x 10^6^, 50) | 0.6 x 10^-2^ | |
| | TAM | ER-α46 | ER-α46+/pAKT+ (1.37 x 10^6^, 30) | ER-α46+/pAKT- (0.90 x 10^6^, 12) | 0.3 x 10^-1^ | |
| | TAM | p38 MAPK | ER-α66+/p38 MAPK+ (1.89 x 10^6^, 138) | ER-α66+/p38 MAPK- (1.38 x 10^6^, 345) | 0.5 x 10^-3^ | |
| | TAM | ER-α66 | ER-α66+/p38 MAPK+ (0.54 x 10^6^, 212) | ER-α66+/p38 MAPK- (0.62 x 10^6^, 12) | 0.7 | |
| | TAM | ER-α66 | ER-α66+/pS6+ (1.13 x 10^6^, 62) | ER-α66+/pS6- (0.56 x 10^6^, 94) | 0.1 x 10^-3^ | |
| | TAM | ER-α46 | ER-α46+/pS6+ (1.73 x 10^6^, 15) | ER-α46+/pS6- (0.95 x 10^6^, 27) | 0.9 x 10^-2^ | |
| MDA-MB-231 | TAM | ER-α46 | ER-α46+/pAKT+ (2.29 x 10^7^, 17) | ER-α46+/pAKT- (1.40 x 10^7^, 56) | 0.1 x 10^-2^ | |
| | TAM | CD44 | ER-α46+/CD44+ (1.79 x 10^7^, 37) | ER-α46-/CD44+ (3.34 x 10^6^, 245) | < 0.1 x 10^-3^ | |
Table S4 TAM effects on MCF-7 and MDA-MB-231 subpopulations expressing ER-α protein targets.
