# Supplementary figures and images for: Single-cell immunoblotting resolves estrogen receptor-α isoforms in breast cancer
Source: PLoS One. 2021 Jul 27;16(7):e0254783. doi: 10.1371/journal.pone.0254783 (PMC8315538; doi:10.1371/journal.pone.0254783)

# HC-20 Blot (S1 Fig)

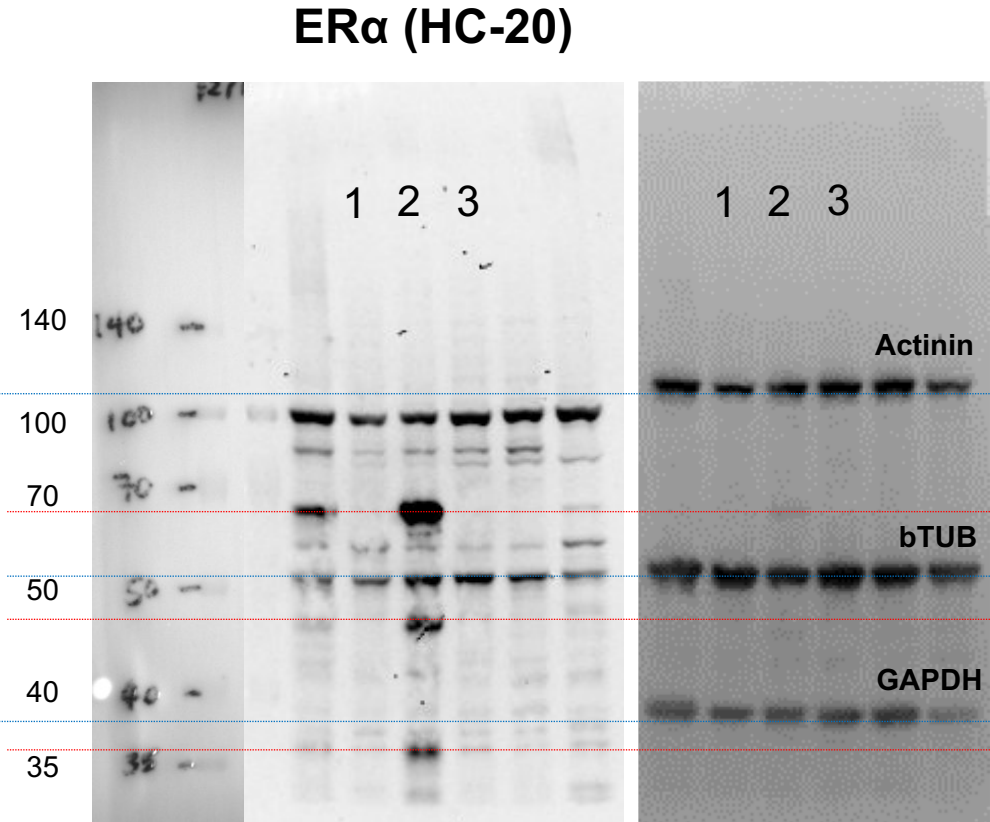

1: HEK293, 2: MCF-7, 3: MDA-MB-231

# H226 Blot (S1 Fig)

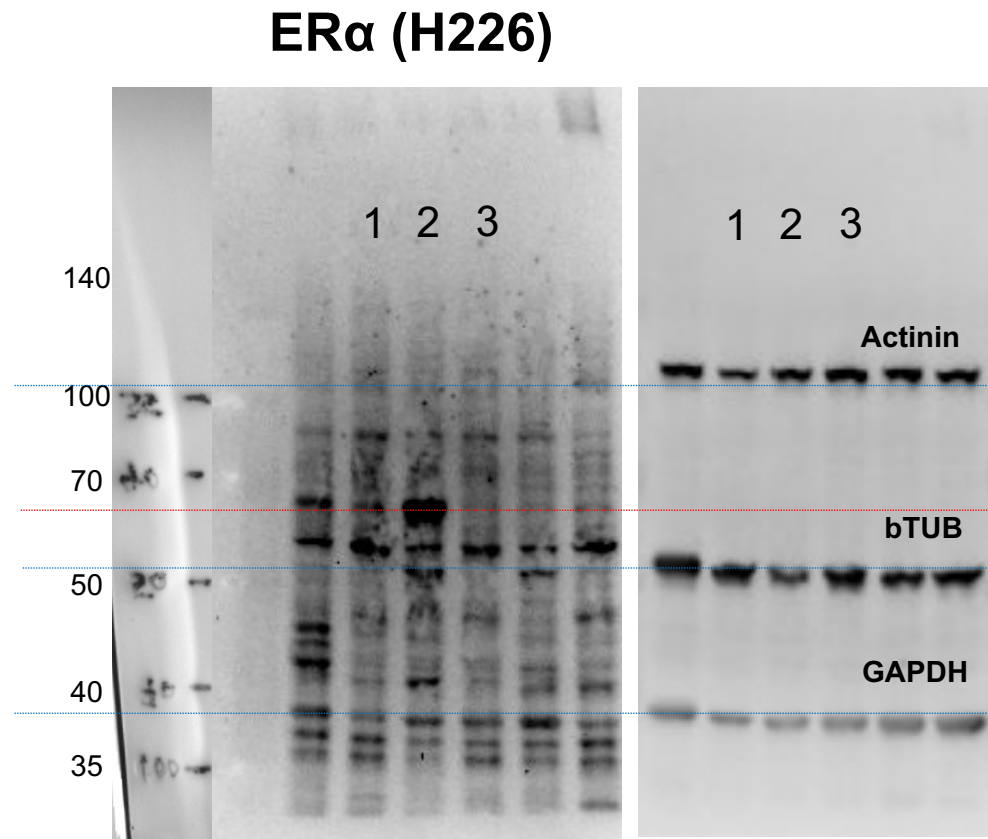

1: HEK293, 2: MCF-7, 3: MDA-MB-231

# SP-1 Blot (S1, S3 Figs)

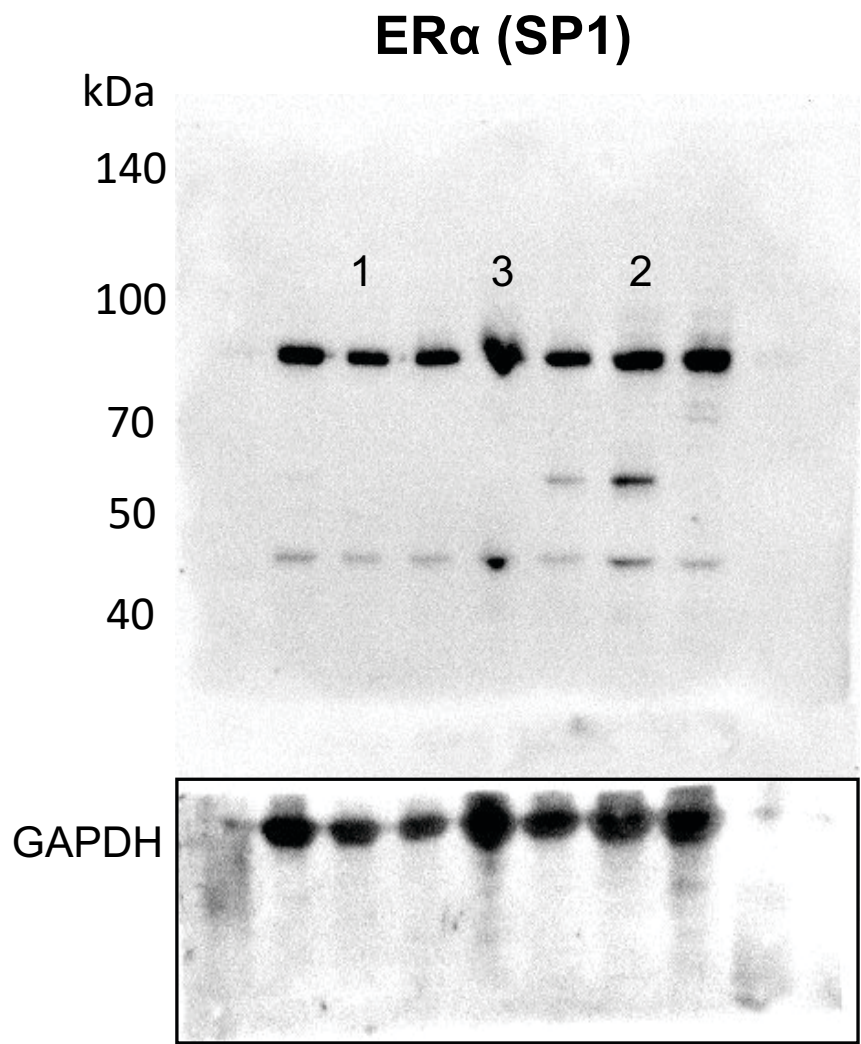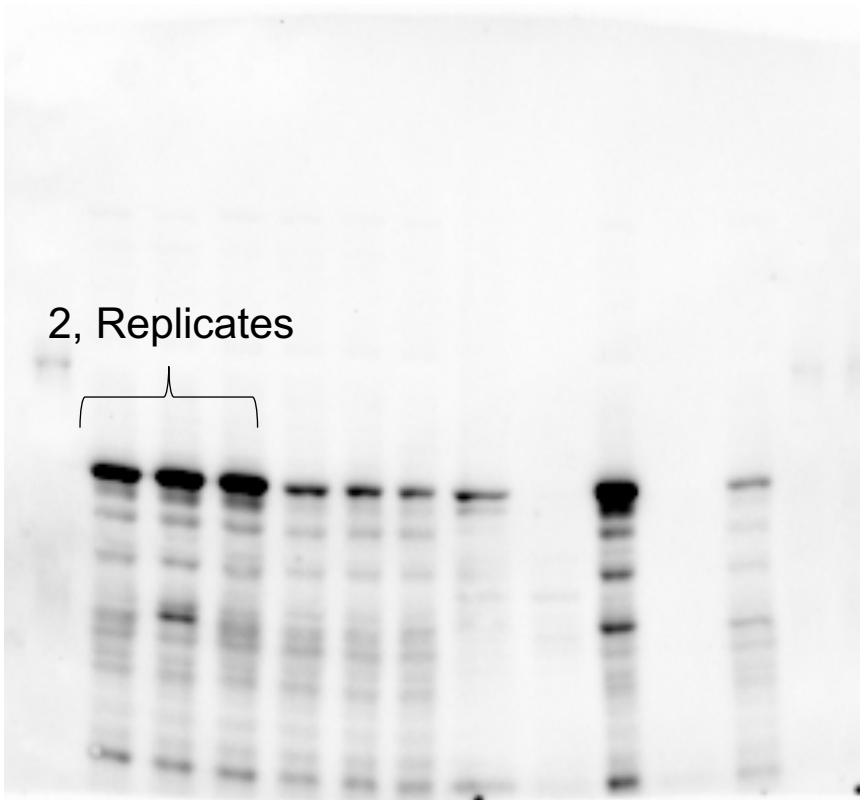

1: HEK293. 2: MCF-7. 3: MDA-MB-231

Supplement: S1 Raw images — (PDF) [file pone.0254783.s002.pdf]
