# Supplementary material for: Single-cell immunoblotting resolves estrogen receptor-α isoforms in breast cancer
Source: PLoS One. 2021 Jul 27;16(7):e0254783. doi: 10.1371/journal.pone.0254783 (PMC8315538; doi:10.1371/journal.pone.0254783)
Supplement: S1 Text — (DOCX) [file pone.0254783.s003.docx]

**TITLE:** Single-cell immunoblotting resolves estrogen receptor-α isoforms in breast cancer

John J. Kim^1^, Wenchuan Liang^2^, Chi-Chih Kang^1^, Mark D. Pegram^2^ and Amy E. Herr^1,*^

^1^Department of Bioengineering, University of California Berkeley, Berkeley, CA 94720, USA

^2^Division of Medical Oncology, Department of Medicine, Stanford University, Stanford, CA 94305, USA

Correspondence should be addressed to Dr. Amy E. Herr (email: [aeh@berkeley.edu](mailto:aeh@berkeley.edu), Tel: 510- 666-3396, Fax: 510-642-5835, <http://herrlab.berkeley.edu>)

**Supporting information**

**Cell lines and cell culture**

HEK293 was obtained from the American Type Culture Collection (ATCC). HEK293 was cultured in Eagle's Minimum Essential Medium (EMEM) (30-2003; ATCC) supplemented with 1% PS and 10% FBS. All cell lines were incubated in a humidified incubator held at 37 ˚C under 5% CO_2_. HEK293 was authenticated using short tandem repeat analysis by UC Berkeley Cell Culture Facility.

**Estradiol**

Prior to ligand treatment, cells were incubated in phenol free RPMI1640 (11835030; Thermo Fisher Scientific) and charcoal stripped FBS (A3382101; Thermo Fisher Scientific) with 1% PS for 48 h. E2 (E8875; Sigma-Aldrich) were dissolved in 100% EtOH and prepared at 1 mM concentration. Then, cells were treated with E2 with final concentration of 1 μM for 24 h. For negative control, cells were treated with 100% EtOH with equal volume as in the E2 treatment for 24 h. After the treatment, cells were detached from cell culture dish with 10 mM EDTA (AM9260G, Thermo Fisher Scientific) and proceeded with the single-cell immunoblotting.

**Slab-gel immunoblotting**

Cells from a 10 cm petri dish at 80% confluency were lysed with a radioimmunoprecipitation assay (RIPA) buffer and centrifuged to collect proteins. Each sample was boiled at 95˚C for 5 min and separated on a 10% SDS-PAGE and transferred on a nitrocellulose membrane using a semi-dry blot machine (Pierce Power Blotter; Thermo Fisher Scientific). The membrane was blocked with 1X TBST buffer with 5% FBS for 1 h. The membrane was incubated with a primary antibody (1:15,000) for overnight at 4 ˚C and washed with 1X TBST buffer for 1 h. The membrane was then incubated with secondary antibodies labelled with HRP for 1 h at room temperature. After washing with 1X TBST for 1 h, the membrane was incubated with HRP chemiluminescent substrate (SuperSignal West Dura Substrate, 34076; Thermo Fisher Scientific) for 5 min and detected with a CCD imager (ChemiDoc XRS+, Bio-Rad).

**Fluorescence-activated cell sorting via flow cytometry**

MCF-7 and MDA-MB-231 cells were collected at 10^6^ cells/ml. The cells were fixed with 4% formaldehyde (12606; Cell Signaling) for 10 min at 37 ˚C. The cells were permeabilized with 90% MeOH for 30 min on ice. After washing the cells with an incubation buffer (1x PBS with 2% FBS) twice, the cells were resuspended in 100 µl of primary antibody (1:50) and incubated at room temperature for 1 h. The cells were washed again with the incubation buffer and incubated with secondary antibody (1:150) for 30 min at room temperature. The cells were washed again with the incubation buffer and analyzed by using Attune NxT Flow Cytometer (Thermo Fisher Scientific). Viable cells were gated on size and shape using forward and side scatters. The cytometer was set at 200 cells/ml. FITC fluorescence was collected in the BL1 channel using a 480/500 nm band-pass filter.

**Validation of ER-α antibodies with breast cancer (BCa) cell lines.**

In order to distinguish ER-α66 and ER-α46, we examined a panel of antibodies with model BCa cell lines in conventional immunoassays. Amino acid sequences of ER-α66 and ER-α46 are homologous except at the N-terminal AF1 transactivation domain **(S2A Fig**) [1]. First, ER-α antibodies targeting N and C terminal domains were validated via slab-gel immunoblotting of lysates pooled from ~10^6^ cells from 3 cell lines: MCF-7 (hormone-sensitive BCa cell line with ER-α66^+^/ ER-α46^+^), MDA-MB-231 (hormone-insensitive BCa cell line with ER-α66^-^), HEK293 (kidney cell line missing ER-α isoforms). Based on molecular sizing, antibodies that detected ER-α66 (66 kDa) and ER-α46 (46 kDa) isoforms were SP-1, H226, and HC-20 antibodies (**S2B Fig**). Out of the three antibodies, SP-1 distinctively revealed ER-α66 and ER-α46 in a MCF-7 lysate with 1 non-specific protein peak at a 100-kDa location, ER-α46 in a MDA-MB-231 lysate, and no ER-α66 in HEK293 (**S2B, S4C Figs**).

We further investigated ER-α selectivity by utilizing immunocytochemistry (ICC) and fluorescence-activated cell sorting (FACS). Because ER-α is a family of nuclear receptors largely present inside a cell nucleus, we found out that SP-1 and HC-20 stain align with nucleus staining (Hoechst 33342) for DNA in MCF-7 (**S2C Fig**). Quantitatively, with respect to HEK293, SP-1 identified 51% of MCF-7 cells expressing ER-α isoforms (**S2D Fig**). After validating ER-α antibodies across conventional immunoassays, we performed single-cell immunoblotting to discern ER-α isoforms.

**Development of polyacrylamide gel electrophoresis for ER-α isoforms**

In the single-cell immunoblot, the *in-situ* cell lysis affects the downstream workflow of protein detection. Consequently, the cell lysis components need to be optimized for protein solubility. As ER-α binds to DNA and resides in the cell nucleus and cytoplasm, we sought detergent components to resolve ER-α66 and ER-α46. In the single-cell immunoblot, detergents affect protein solubility, thereby influencing protein separation resolution. We tested sodium dodecyl sulfate (SDS), an anionic detergent that disrupts protein membranes and denatures proteins, at 1% and 2% (m/v) concentrations for the cell lysis. Unexpectedly, increasing SDS concentration from 1% to 2% decreased separation resolution between ER-α66 and GAPDH from 0.73 to 0.41 (**S4A Fig**). Another component we investigated to optimize cell lysis conditions was a chaotrope, urea. Urea is commonly incorporated in cell lysis to denature proteins by disrupting hydrogen bonds and lowering detergent critical micelle concentration [2,3]. In the single-cell immunoblot, the inclusion of 8M urea in the lysis buffer improved separation resolution of ER-α66 and GAPDH from 0.62 to 1.72 without diminishing SNR (**S4A Fig**).

After optimizing the lysis buffer conditions with 1% SDS and 8M urea, we tested the three ER-α antibodies in the single-cell immunoblot workflow (SP-1, H226, and HC20) to identify ER-α isoforms in MCF-7, MDA-MB-231, and HEK293 cells (**S4B Fig**). The H226 and HC20 antibodies generated non-specific peaks in HEK293 and MDA-MB-231 cells, respectively (**S4B Fig**). In contrast, the SP-1 antibody distinguished ER-α66 and ER-α46 in the MCF-7 cells, without producing non-specific signals in any of the cell lines (**S4B Fig**). Hence, we chose the SP-1 antibody to investigate the frequency and expression levels of ER-α isoforms in BCa cell lines.

**Single-cell immunoblot analysis of MCF-7 and MDA-MB-231 populations**

Before investigating interactions between ER-α isoforms and the target proteins in the ER-α pathways, we initially sought to find TAM effects on each protein target at a population level. In MCF-7, pAKT, p38 MAPK, ER-β are three canonical-target proteins that respond to TAM. Moreover, TAM repressed mean pAKT expression level (**S2 Table, S6A Fig**). We did not find any significant TAM effects on EGFR (**S2** **Table, S6A Fig**). In the non-canonical pathway, mean cJUN expression level was enhanced by TAM. We did not find significant changes in mean CD44, Cyclin A, and pS6 expression levels at the MCF-7 population level. In contrast to MCF-7, we found distinctive protein signatures in MDA-MB-231. E2 further repressed mean pS6 expression level (**S2** **Table, S6B Fig**). TAM induced mean Cyclin A expression level while inhibited p38-MAPK and ER-β (**S2 Table, S6B Fig**).

**SI References**

1. Chantalat E, Boudou F, Laurell H, Palierne G, Houtman R, Melchers D, et al. The AF-1-deficient estrogen receptor ERα46 isoform is frequently expressed in human breast tumors. Breast Cancer Res. 2016;18. doi:10.1186/s13058-016-0780-7

2. Peach M, Marsh N, Miskiewicz EI, MacPhee DJ. Solubilization of Proteins: The Importance of Lysis Buffer Choice. In: Kurien BT, Scofield RH, editors. Western Blotting: Methods and Protocols. New York, NY: Springer New York; 2015. pp. 49–60. doi:10.1007/978-1-4939-2694-7_8

3. Nalvarte I, Schwend T, Gustafsson J-A. Proteomics analysis of the estrogen receptor alpha receptosome. Mol Cell Proteomics. 2010;9: 1411–22. doi:10.1074/mcp.M900457-MCP200
